# Analysis of an optimal hidden Markov model for secondary structure prediction

**DOI:** 10.1186/1472-6807-6-25

**Published:** 2006-12-13

**Authors:** Juliette Martin, Jean-François Gibrat, François Rodolphe

**Affiliations:** 1 INSERM U726, Equipe de Bioinformatique Génomique et Moléculaire Université Denis Diderot Paris 7, 2 place jussieu, 75251 Paris Cedex 05, France; 2 INRA, Unité Mathématiques Informatique et Génome, Domaine de Vilvert, 78352 Jouy en Josas Cedex, France

## Abstract

**Background:**

Secondary structure prediction is a useful first step toward 3D structure prediction. A number of successful secondary structure prediction methods use neural networks, but unfortunately, neural networks are not intuitively interpretable. On the contrary, hidden Markov models are graphical interpretable models. Moreover, they have been successfully used in many bioinformatic applications. Because they offer a strong statistical background and allow model interpretation, we propose a method based on hidden Markov models.

**Results:**

Our HMM is designed without prior knowledge. It is chosen within a collection of models of increasing size, using statistical and accuracy criteria. The resulting model has 36 hidden states: 15 that model *α*-helices, 12 that model coil and 9 that model *β*-strands. Connections between hidden states and state emission probabilities reflect the organization of protein structures into secondary structure segments. We start by analyzing the model features and see how it offers a new vision of local structures. We then use it for secondary structure prediction. Our model appears to be very efficient on single sequences, with a Q3 score of 68.8%, more than one point above PSIPRED prediction on single sequences. A straightforward extension of the method allows the use of multiple sequence alignments, rising the Q3 score to 75.5%.

**Conclusion:**

The hidden Markov model presented here achieves valuable prediction results using only a limited number of parameters. It provides an interpretable framework for protein secondary structure architecture. Furthermore, it can be used as a tool for generating protein sequences with a given secondary structure content.

## Background

Predicting the secondary structure of a protein is often a first step toward 3D structure prediction of a particular protein. In comparative modeling, secondary structure prediction is used to refine sequence alignments, or to improve the detection of distant homologs [1]. Moreover, it is of prime importance when prediction is made without a template [2]. For all these reasons protein secondary structure prediction has remained an active field for years. Virtually all statistical and learning methods have been applied to this task. Nowadays, the best methods achieve prediction rate of about 80% using homologous sequence information. A survey of the Eva on-line evaluation [3] shows that the top performing methods include several approaches based on neural networks, e.g. PSIPRED by Jones et al [4], PROFsec and PHDpsi by Rost et al [5]. Recently several publications reported secondary structure prediction using SVM [6-8]. A number of attempts using Hidden Markov Models (HMM) have also been reported. A particularity of these models is their ability to allow an explicit modeling of the data. The first attempt to predict secondary structure with HMMs was due to Asai et al [9]. Asai et al presented four sub-models, trained separately on pre-clustered sequences belonging to particular local structures: alpha, beta, coil and turns. The sub-models, each of them made of four or five hidden states, were then merged into a single model, achieving a *Q*_3 _score of 54.7%. At the same period, Stultz et al [10,11] proposed a collection of HMMs representing specific classes of proteins. The models were "constructed as generalization of the study-set example structures in terms of allowed connectivities and surface loop/turn sizes" [10]. This involved the distinction of N-cap and C-cap positions in helices, an explicit model of amphipatic helices and *β*-turns. Each model being specific of a protein class, the method required first that the appropriate hidden Markov model be selected and then used to perform the secondary structure prediction. The *Q*_3 _scores, reported for only two proteins, were respectively 66 and 77%. Goldman et al [12-15] proposed an approach unifying secondary structure prediction and phylogenetic analysis. Starting with an aligned sequence family, the model was used to predict the topology of the phylogenetic tree and the secondary structure. The main feature of this model was the inclusion of the solvent accessibility status, and the constrained transitions to take into account the specific length distribution of secondary structure segments. The *Q*_3 _score, reported for only one sequence family, was 65.7% using single sequence and 74.4% using close homologs. Later, Bystroff et al [16] proposed a complex methodology based on the I-Sites fragment library. One of the models was dedicated to the prediction of secondary structures. The model construction made use of a number of heuristic criteria to add or delete hidden states. The resulting models were quite complex and modeled the protein 3D structures in term of succession of I-site motifs. The prediction accuracy of the model dedicated to secondary structure prediction was 74.3%, using homologous sequence information. Other approaches used slightly different type of HMM, based on the concept of a sliding window along the secondary structure sequence. Crooks and Brenner [17] proposed a methodology where a hidden state represents a sliding window along the sequence. The prediction accuracy was 66.4% for single sequences and 72.2% with homologous sequence information. Zheng et al [18] used a similar approach in combination with amino-acid grouping, achieving a *Q*_3 _score of 67.9% on single sequences. An extension of hidden Markov Model, semi-HMM, were also applied to secondary structure prediction by several groups [19-21]. These models allow an explicit consideration of the length of secondary structures. Very recently, Aydin et al claimed a *Q*_3 _score of 70.3% for single sequences [22]. Chu et al [21] obtained a *Q*_3 _score of 72.8% using homogous sequence information.

Here, we exploit a novel HMM learned from secondary structures without taking into account prior biological knowledge [23]. Because the choice of a particular model does not rely on any prior constraints, the HMM itself is an interesting tool to reveal hidden features of the internal architecture of secondary structures. We first analyze in detail the model. We then evaluate its predictive potential on single sequences and on multiple sequence information using an evaluation data set of 506 sequences and a data set of 212 sequences obtained from the EVA Web site [24]. The influence of the secondary structure assignment method on the performance is also discussed. The prediction results appear very promising and open the perspective for further refinements of the method.

## Results and discussion

### Hidden Markov model selection

The optimal hidden Markov model for secondary structure prediction, referred as OSS-HMM (Optimal Secondary Structure prediction Hidden Markov Model), was chosen using three criteria: the *Q*_3 _achieved in prediction, the Bayesian Information Criterion (BIC) value of the model and the statistical distance between models. The whole selection procedure described in details in [23]. Here we only present the main steps of the selection. Let *n*_*H*_, *n*_*b *_and *n*_*c *_be the number of hidden states that model respectively *α*-helices, *β*-strands and coils. The optimal model selection was done in three steps. In the first step, we set *n*_*H *_= *n*_*b *_= *n*_*c *_= *n *and models were estimated with *n *varying from 1 to 75. The *Q*_3 _score evolution indicated that 10 to 15 states were sufficient to obtain good prediction level without over-fitting and that increasing *n *above 15 had little impact on the prediction. The BIC selected a model with *n *= 14. The statistical distances between models revealed a great similarity across models for *n *varying from 13 to 17. In the second step, models were thus estimated with (1) *n*_*H *_= 1 to 20 and *n*_*b *_= *n*_*c *_= 1, (2) *n*_*b *_= 1 to 15 and *n*_*H *_= *n*_*c *_= 1 or (3) *n*_*c *_= 1 to 15 and *n*_*H *_= *n*_*b *_= 1. The BIC selected (1) *n*_*H *_= 15, (2) *n*_*b *_= 8 and (3) *n*_*c *_= 9. In the third step, all the architectures were tested with *n*_*H *_varying from 12 to 16, *n*_*b *_from 6 to 10 and *n*_*c *_from 3 to 13. The BIC selected the optimal model having *n*_*H *_= 15, *n*_*b *_= 9 and *n*_*c *_= 12, i. e., a total of 36 hidden states. Overall, nearly 300 different model architectures were tested in this procedure [23]. The automatic generation of a HMM topology has been previously addressed by several groups [25-29]. Two main strategies were used: the first one consists in building models of increasing size starting from small models [25,29] and the second one, inversely, consists in progressively reducing large models [26,28]. Regarding the first strategy, the use of genetic algorithm was introduced and applied to the modeling of DNA sequences [25,29]. In the approach presented by Won et al [29], an initial population of hidden Markov models with 2 states is submitted to an iterative procedure of mutations, training and selection. There are five types of mutation: addition or deletion of one hidden state, addition or deletion of one transition and cross-over, consisting in exchanging several states between two HMMs. The second strategy requires the use of a pruning algorithm and was applied to language processing [26] and to describe the structure of body motion data [28]. The initial model, consists of an explicit modeling of the training data: each sample is represented by dedicated HMM states. Hidden states are then removed iteratively by merging two states. The merging criterion is based on the evolution of the log-likelihood.

Our approach of automated selection of HMM topology is related to the first strategy because we also start from small models and increase them afterward. However, Won et al note that their mutation operations, even if they do allow highly connected models, bias the architectures toward chain structures [29]. A previous experience of knowledge-based design of HMM for secondary structure prediction [30] convinced us that highly connected models are more appropriate in our case. We adopt a systematic approach, when we introduce a new state all transitions between hidden states are initially allowed. We then let the system evolve. Our final model does not exhibit a chain structure. A major concern of the genetic algorithm applied to HMM topology seems to be the over-fitting of the model toward learning data [25,29]. In our approach, the over-fitting is monitored by considering an independent set of structures that is never used in the cross-validation procedure. An original aspect of our method is that we not only check the fit with the model but also the predictive capabilities on these independent data. The other strategy described in the literature based on the merging of states requires the manipulation of large initial models. The large size of the learning datasets available for secondary structure prediction might be a problem when employing such a strategy. Nonetheless, a common feature of our approach with the work of Vasko et al [28] is the use of initial models were all transitions are allowed.

It is likely that the appropriate strategy for automated topology selection depends on the amount and nature of data to be modeled. Here we are confronted with a problem with large datasets and, presumably, a complex connected underlying structure. Our approach results in a model having a reasonable number of parameters although it is larger and more complex than the one we designed in a knowledge-based approach [30].

### Hidden Markov model analysis

Our optimal model OSS-HMM was built without prior knowledge and without imposing any constraint. Thus, it is interesting to examine the final model as it reveals the internal architecture of secondary structures learned by the model. In the following section, we will describe the main features of the model obtained with DSSP assignment and link these observations with previous studies.

All transitions between hidden states are initially allowed. As shown in Table 1, many transitions in the final model are estimated to have probability zero. Only 36% of potential transitions remain within the helix box, 57% within the strand box and 68% in the coil box. This highlights a first feature of secondary structures: even though helices require 15 hidden states they can be modeled by relatively few transitions. On the contrary, *β*-strand sequences are fuzzier, with a higher connectivity between hidden states. Thus, the paths within the helix box are more constrained than in the strand box. The final model has 448 non-null transitions (out of 1296), of which 89 have a probability greater than 0.1, for a total of 1096 free parameters.

**Figure 1:**
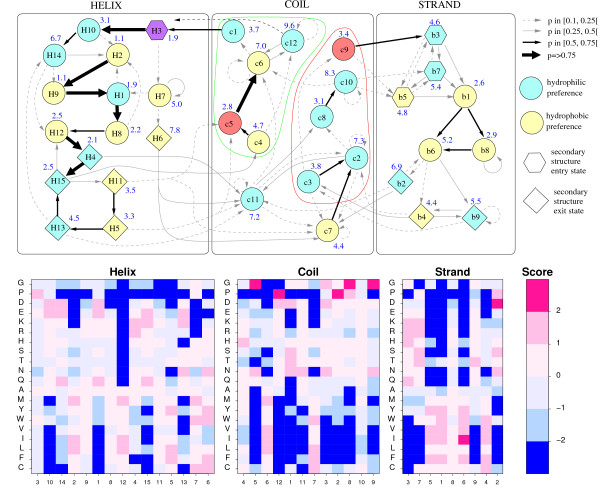
**Final 36 hidden states HMM learned using DSSP assignment**. Upper part: hidden state graph. Only transitions associated with probabilities greater than 0.1 are shown. The larger the transition probabilities the thicker the arrows. States are colored according to their amino acid preference (hydrophobic versus hydrophilic). Purple state indicates no strong amino acid preference and red states strongly favor glycine. The two groups of coil states (c1, c6, c12, c5, c4) in green and (c3, c2, c8, c10, c9) in red are discussed in the text. For periodic secondary structures, helix and strand, the entry and exit states are indicated by different symbols. Lower part: amino-acid propensities of each hidden state. Propensities are measured by log-odd scores. The propensity score of amino-acid *a *for state *s *is given by: S=log2P(a|s)f(a)
 MathType@MTEF@5@5@+=feaafiart1ev1aaatCvAUfKttLearuWrP9MDH5MBPbIqV92AaeXatLxBI9gBaebbnrfifHhDYfgasaacH8akY=wiFfYdH8Gipec8Eeeu0xXdbba9frFj0=OqFfea0dXdd9vqai=hGuQ8kuc9pgc9s8qqaq=dirpe0xb9q8qiLsFr0=vr0=vr0dc8meaabaqaciaacaGaaeqabaqabeGadaaakeaacqWGtbWucqGH9aqpieGacqWFSbaBcqWFVbWBcqWFNbWzdaWgaaWcbaGaeGOmaidabeaakmaalaaabaGaemiuaaLaeiikaGIaemyyaeMaeiiFaWNaem4CamNaeiykaKcabaGaemOzayMaeiikaGIaemyyaeMaeiykaKcaaaaa@3F9E@, with *P*(*a *| *s*) the emission probability of amino-acid *a *in state *s *and *f*(*a*) the background frequency of *a *in the dataset. A score equal to 1 means that the amino-acid is twice as frequent in state *s *as in the whole dataset.

The structure of OSS-HMM is presented in Figure 1. For the sake of clarity, only transitions with probabilities larger than 0.1 are shown. Hidden states are colored according to their amino acid preference. The blue figure close to each state is its *Neq *value. The *Neq *value of a hidden state is an estimation of the number of output states, derived from the Shannon Entropy:

Neq(s)=exp⁡[∑r−p(s;r)ln(p(s;r))]
 MathType@MTEF@5@5@+=feaafiart1ev1aaatCvAUfKttLearuWrP9MDH5MBPbIqV92AaeXatLxBI9gBaebbnrfifHhDYfgasaacH8akY=wiFfYdH8Gipec8Eeeu0xXdbba9frFj0=OqFfea0dXdd9vqai=hGuQ8kuc9pgc9s8qqaq=dirpe0xb9q8qiLsFr0=vr0=vr0dc8meaabaqaciaacaGaaeqabaqabeGadaaakeaacqWGobGtcqWGLbqzcqWGXbqCcqGGOaakcqWGZbWCcqGGPaqkcqGH9aqpcyGGLbqzcqGG4baEcqGGWbaCcqGGBbWwdaaeqbqaaiabgkHiTiabdchaWjabcIcaOiabdohaZjabcUda7iabdkhaYjabcMcaPGqaciab=XgaSjab=5gaUjabcIcaOiabdchaWjabcIcaOiabdohaZjabcUda7iabdkhaYjabcMcaPiabcMcaPiabc2faDbWcbaGaemOCaihabeqdcqGHris5aaaa@524C@

where *p*(*s*; *r*) denotes the transition probability from state *s *to state *r*. The sum is taken over all the hidden states. *Neq *varies from 1 (only one output state) to the total number of hidden states (a state uniformly connected to all the others, including itself). Emission parameters are also presented in the lower part of Figure 1. The emission probability of each amino-acid in each hidden state is given using preference score relative to amino-acid background frequencies.

Some general remarks can be made from this representation:

• The different secondary structures are characterized by different transition usage. Very strong transitions appear in the helix box, whereas they are weaker in strand and coil boxes. This is confirmed for helices since some helical states have very low *Neq*: e. g., H3, H2, H9 and H1 have Neq lower than 2, meaning that the number of output states is limited. No such states are apparent in the strand and coil boxes. The mean *Neq *value per state is 3.28 for helix, 4.70 for strand and 5.44 for coil. This confirms the observations made from Table 1: helices appear very "structured" motifs with strong transitions and few paths allowed, whereas strands appear to be "fuzzier", less constrained, with many alternative paths and relatively low transitions probabilities. The coil box is even less structured than the strand box.

• An examination of amino acid preference scores shows that hidden states specifically tend to *avoid *particular residues (many scores less than -2), rather than *favor *other ones (few scores greater than 2). This seems to denote a kind of "negative design", where there is a stronger constraint *not to include *particular residues, than *to have *some others.

#### Helix architecture

Helices in proteins are characterized by the so-called amphipathic rule (also known as helical wheel rule): one face is in contact with the solvent and thus bears hydrophilic residues and one face is in contact with the protein interior and shows preference for hydrophobic residues. The canonical *a *helix has a periodicity of 3.6. The helix periodicity and the amphipathic rule generate a periodicity of 3 or 4 in terms of amino acid properties in the sequence.

The helix box is characterized by a topology that allows unidirectional paths through the graph.

• There is only one entry state for helices: state H3. The amino acid preference of this state is peculiar since it does not favor nor disfavor any amino acids except for a slight tendency to prefer proline and to avoid asparagine. Only state c10 shows a similar lack of strong preference for amino acids (i.e., without score larger than 2 or smaller than -2 and only two scores greater than 1 or less than -1).

• Two alternative trajectories among the states are then possible: a "bypass" trajectory proceeding to state H7 and exiting the helix at state H6 and the "main" trajectory that is detailed below. Note that there is a small probability to get back to the main trajectory from the bypass trajectory (state H7). Interestingly this state, H7, is the only helix state with a self transition.

• States belonging to the main trajectory can be divided into two groups: core states (H10, H14, H2, H9, H1, H8, H12) and exit states (H4, H15, H11, H5, H13). As expected from the helix periodicity and amphipathic rule, the graph shows a mixture of 3-state and 4-state cycles with characteristic patterns of hydrophobic and hydrophilic preferences. It is likely that the strong directionality observed between the core states is due to the fact that once in a helix the system must remain in this helix for at least 4 or 6 residues, i.e., one turn or one turn and a half. In other words, the core states correspond to the beginning of a helix.

• The second group of states, the exit states, shows the same 3-state and 4-state cycles with similar patterns of hydrophilic or hydrophobic preferences as the core states but now the cycles can be interrupted at any time to move to a coil state. This shows that helices can end without the need for completing a turn.

#### Strand architecture

The strand box shows a different structure. The progression through the graph is also rather directional:

• There are 3 entry states (b3, b5, b7) that are all interconnected. All entry states are also connected to the same state, b1, that belongs to the core of the strand architecture.

• the core of the strand architecture is made of states b1, b6, and b8 that form a 3-state cycle. State b1 is connected to entry states, state b6 to exit states and state b8 is one of the two strand states that exhibit a self transition. It is interesting to note that the core of the strand architecture contains only states with a preference for hydrophobic amino acids.

• There are 3 exit states, b2, b4 and b9. There exists two exit routes, one through b2 that is connected to state c7 and c11 and one through states b9 and b4 that are connected to states c3 and c2. b2 is peculiar in that, unlike other strand states, it favors proline and aspartate.

There is a number of 2-state cycles that correspond to an alternation of hydrophilic and hydrophobic states. This is a known pattern, observed in strands that are at the protein surface. Another pattern often observed in strands is the occurrence of several hydrophobic residues. This pattern is represented by paths amongst the core states b1, b8 and b6. It is also known that hydrophobic/hydrophilic patterns in strands are fuzzier than similar patterns in helices. This is clearly apparent when one compares the strength of the connections between states in the helix and strand boxes.

When assigning secondary structure with DSSP, there are very few helices immediately followed by a strand or strands immediately followed by a helix therefore direct connections between helices and strands are associated with small probabilities that do not appear on Figure 1.

#### Coil architecture

The graph structure of the coil box presents a very different organization compared to the helix and strand boxes. The most striking feature is the existence of specific states leading to and coming from helices or strands (there are only two "core" states, c8 and c6, that are exclusively connected to other coil states). Unlike the two other boxes, half the states in the coil box (c2, c6, c7, c10, c11, c12) have self transitions. The coil states can be divided into 3 groups:

• states c11 and c7 are the only states that are found both at the termination of helices and strands (although only the exit route through b2 leads to this states). These states are very connected, including a self connection and act as kinds of "hub" in the coil box.

• a group of states (c2, c3, c8, c10, c9) within the red contour that interact with strands.

• a group of states (c4, c5, c6, c1, c12) within the green contour that interact with helices.

These two groups have the same organization: two states are connected to secondary structure exit states (c4 and c5 after a helix exit state and c2 and c3 after a strand exit state), one state is a "core" state, c6 for the first group and c8 for the second group, and two states lead to a secondary structure entry state (cl and c12 to the helix entry state, and c9 and c10 to the strand entry states). These two groups are relatively independent, there is a weak direct connection between the two groups (through states c10 and c6) and an indirect connection through the hub state c11. These two groups seems to model different types of loop. To further analyze the specific usage of the states in different loop types, the paths through the coil states are analyzed for different kind of loops. Note that these paths are obtained with the Viterbi algorithm. Both amino-acid and secondary structure sequences are given as input to OSS-HMM: in a way similar to the estimation of labeled sequence of secondary structures [31], it is possible to monitor the Viterbi algorithm using the product of amino-acid and label emission parameters instead of the amino-acid emissions only. The protein sequence and the label sequence are considered to be independent emissions by the same hidden process. In that case, the Viterbi algorithm computes the most probable trajectory in the hidden Markov Model, given the real secondary structure of the protein. Hence, it is not a secondary structure prediction, but the analysis of protein sequences using OSS-HMM. We then collect the number of times each coil state is observed in the four different classes of loops: *α*/*α*, *β*/*β*, *α*/*β *and *β*/*α*. The data obtained on the cross-validation set are shown in Table 2.

**Figure 2:**
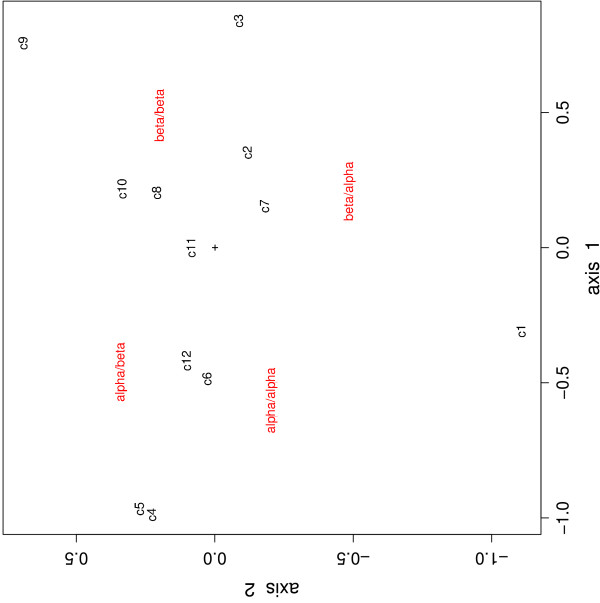
**Principal component analysis of the association between hidden states and loop type**. Data are obtained from the Viterbi decoding using secondary structure labeling on the cross-validation data.

The repartition of the coil states indicates that the hidden states are preferentially found in particular loop types. For example, state c6 is observed 5256 times in *a/a *loops and only 2894 in *β*/*β *loops, although *α*/*α *loops represent 20.1% of the data, and the *β*/*β *loops represent 35%. To take into account this non-uniform repartition of different loop types, Table 2 is then submitted to a correspondence analysis. The data projection on the first two axes is shown on Figure 2. The first two axes respectively explain 66% and 32% of the variance. As shown by Figure 2, the first axis differentiate loops found after an helix (*α*/*α *and *α*/*β*) on one hand, and loops found after a strand (*β*/*β *and *β*/*α*) on the other hand. It means that, according to the hidden state usage, *α*/*α *or *α*/*β *differ from *β*/*β *or *β*/*α *loops. The data projection confirms the observations made from Figure 1: states c4, c5, c6 and c12 appear clearly associated to *α*/*α *loops and states c2, c3, c8, c9 and c10 to *β*/*β *loops. When projected on the first axis, state c1 appears close to the *α*/*α *type.

As shown on Figure 1 states c11 and, to a lesser extend c7, act as "hubs", allowing to switch between *α*/*α *and *β*/*β *loops. They are located near the origin on the correspondence plot. States of the coil box show a marked tendency to favor particular amino acids: glycine for states c5, c8 and c9 and proline for states c12, and c2.

#### Comparison with available data in the literature

Several authors studied the sequence specificities of helix termini [32-35]. A direct comparison with these previous studies is not straightforward for several reasons. First, secondary structure boundaries must be the same. As we showed in a previous study [36], the boundaries of helices and strands are the main point of disagreement between different secondary structure assignment methods. For instance, in their study Kumar and Bansal [33] re-assigned helix boundaries (based on DSSP assignment) using geometrical criteria. Thus, the reader should keep in mind that eventual discrepancies might occur because the reference assignment is different (see for example ref. [35] that addresses this particular problem). Secondly, these previous studies often revealed some general trends, whereas our model found several states for helix termination. The computation of general trends leads to the attenuation of the information because they average different signals.

Previous studies [32-35] showed that amino-acid preferences vary according to the position (begin/middle/end) in helices. Our model shows a directional progression in the hidden state graph in agreement with this feature. Aurora and Rose [32] found that PQDE are preferred at the first positions of alpha-helices. The helix starter H3 in our model shows a preference for proline residues, and H10, the second state in helix favors residues D and E. Kumar and Bansal identified a clear preference for residues GPDSTN at positions preceding a helix [33]. It was confirmed in a recent study [35] in which Gibbs-sampling and helix shifting were used to reveal amino-acid preference in conjunction with a potential re-definition of helix termini. The idea was to allow a shift in the helix assignment, to maximize the sequence specificity of helix-cap. In our model, the pre-helix state c1 favors residues D, S, T and N (weakly G). State c12, that also leads to the helix box, has a strong preference for Proline.

A typical motif of C-terminal capping in helices is the termination of an helix by a glycine residue [37]. This feature has also be learned by OSS-HMM, since state c5, that follows an helix, shows a strong preference for glycine. More precisely, Aurora and Rose identified several structural motifs of helix capping, corresponding to distinct sequence signatures [32]. This is in agreement with OSS-HMM since several helix states with different features (e.g. H4 and H6) can terminate an helix, and lead to several coil states (c11, c5, c4).

Engel and DeGrado showed a very clear periodicity of amino-acid distribution in helix cores: alternation of residues with opposite physico-chemical properties every 3 or 4 residues [34]. This corresponds to the well-known model of amphipatic helices. Such helices are found at the interface between protein core and protein surface. One face, thus, bears polar residues and the other face hydrophobic ones. Several cycles appear in the helix core in OSS-HMM: H2/H9/H1 (with possibly H8), H12/H4/H15 and H15/H11/H5/H13. The succession of H12 (hydrophobic), H4 (polar) and H15(polar) fits well with the amphipatic model, as well as the succession H15(polar), H11 (hydrophobic), H5(hydrophobic) and H13(polar). Although in our model, amino-acids are independent, the periodicity of helices has been learned by the model, *via *the hidden structure.

Preferences at strand ends have not been well characterized in the literature. Interestingly, OSS-HMM revealed alternate polar (b3, b7) or apolar (b5) starters of *β*-strands, as well as alternate polar (b2, b9) or apolar (b4) terminators. This could explain why sequence signatures are weak for strand-capping. If several capping motifs with opposite sequence signature co-exist, a global study would conclude to the lack of signature, because several signals are averaged. Our model exhibits several two state cycles that allows the alternation of hydrophobic and polar residues. This alternation has been pointed out in previous studies [38], it reflects the alternation of polar/non-polar environments of residues in *β*-strands at the protein surface.

The path through the four states c3, c2, c8 and c10 allows the connection between two strands. Amino-acid preferences are respectively (G, D, N), (P), (G) and (no preference). The propensities of c3, c2 and c8 fit well with the overall turn potentials identified by Hutchinson and Thornton [39] at position *i*, *i *+ 1 and *i *+ 2 of turns. However, state c10 has no clear amino acid preference.

The correspondence analysis indicates that some states are preferentially used in certain loop type. Previous studies have also revealed that sequence signatures differ according to the loop type [40]. Much like the hydrophobic/polar alternation in amphipatic helices, HMMs can take into account such non strictly local correlations, by specializing hidden states according to the hidden states to which they lead, instead of the amino-acids they emit, and the resulting amino-acid sequence bears non strictly local correlations. Amino-acid emissions are independent but they are conditioned with respect to the hidden process. OSS-HMM provides a new framework for the analysis of protein sequences: for example, the paths within the hidden Markov model could be used to cluster the sequences. It would be particularly interesting to correlate the path based classification with known properties of the structures such as SCOP folds. Preliminary results indicate however that a straightforward classification of the paths is not sufficient [see Additional file 5].

### Use of OSS-HMM for generating protein sequences

Hidden Markov models can be used to generate amino acid sequences that are compatible with the underlying model. OSS-HMM is thus useful in bioinformatics application where simulated protein sequences are needed. For example, in protein threading analysis it is necessary, in order to assess the score significance, to use protein sequences to obtain an empirical score distribution [41].

A simulation study was carried on using OSS-HMM, indicating that simulated sequences share similar amino-acid composition with real protein sequences [see Additional file 3]. Concerning the length distribution of secondary structure elements, as no explicit constraint is integrated in the model, simulated sequences contain more very short segments than real sequences. More sophisticated models, i. e. semi-HMM, are needed to allows an explicit modeling of the length of stay in the hidden states (Aydin et al [22]).

### Secondary structure prediction with OSS-HMM

#### Evaluation on the independent test set

The prediction performance of OSS-HMM is evaluated on an independent test set of 505 protein sequences that were never used for training or model selection. These sequences share no more than 25% sequence identity with the sequences of the cross-validation data set and between sequence pairs; they constitute an appropriate evaluation data set. Prediction is done using a single sequence as input. To compare with existing methods, prediction are also done with the PSIPRED program (version 2.45) [4], using the single-sequence mode. This method is based on neural networks and sequence profiles generated by PSI-BLAST. Here, PSI-BLAST is not used.

Prediction scores obtained with OSS-HMM and PSIPRED are presented in Table 3. Here, the *Q*_3 _score is computed on a *per-residue *basis. The *Q*_3 _score obtained by OSS-HMM is 67.9% and 66.8% with PSIPRED. Due to its limited number of parameters, OSS-HMM exhibits no over-fitting: during the cross-validation, we obtained a mean *per-residue Q*_3 _score of 67.9% on the data used to train the model, and 67.6% on the data not used to train the model (data not shown).

As can be seen from the various scores reported, OSS-HMM is very efficient concerning *α*-helix prediction, with a MCC of 0.56, but less efficient for *β*-strand and coil prediction, with MCC equal to 0.47. The low *Q*_*obs *_(sensitivity) of *β *prediction, 51.9%, and high *Q*_*obs *_for coil prediction, 73.2%, indicate that coils are frequently predicted instead of *β*-strands. This difficulty to predict *β*-strands is a known problem of secondary structure prediction method, probably due to the non-local component of *β*-sheet formation. It is difficult, with a HMM, to take into account this kind of long-range correlation with a model that has a reasonable number of parameters. We tried to integrate a non-local information in the prediction, without any significant amelioration (data not shown).

Comparison with PSIPRED prediction scores shows that OSS-HMM offers a global *Q*_3 _score of more than one point above PSIPRED score (67.9 *vs *66.8%). Such a small difference has to be statistically tested for significance. Since the variances of *Q*_3 _scores per protein obtained by OSS-HMM and PSIPRED were not equal, as assessed by a F-test, the Welch test was used to compare them. The test is significant at the 5% level, we then conclude that OSS-HMM is more efficient than PSIPRED for the single-sequence based prediction. The detailed scores indicate OSS-HMM is better than PSIPRED for helix prediction, both in sensitivity and selectivity. PSIPRED detects more *β*-strand – *Q*_*obs *_is 56.6%, *vs *51.9% for OSS-HMM – but is thus less specific – *Q*_*pred *_is 60.8 *vs *64.5% for OSS-HMM.

#### Testing against EVA dataset

We also tested the prediction accuracy on a dataset of 212 protein sequence from the EVA website. This dataset has been used to rank several secondary structure prediction methods and was the largest available at this time. Using homologous sequence information, the top-three methods on this dataset were:

1. PSIPRED [4]: *Q*_3 _= 77.8%,

2. PROFsec (B. Rost, unpublished): *Q*_3 _= 76.7%,

3. PHDpsi [5] : *Q*_3 _= 75%.

**Figure 3:**
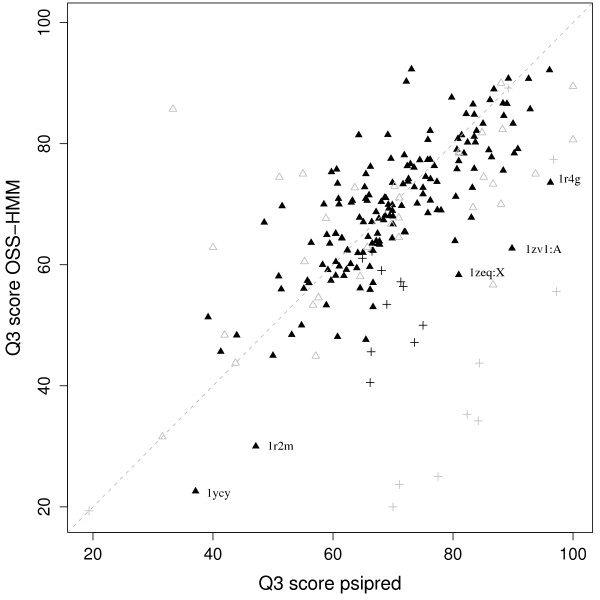
***Q*_3 _obtained for each protein of the EVA 212 dataset, by PSIPRED and OSS-HMM**. Globular proteins are shown as triangles and membrane proteins as crosses. Proteins shorter than 50 residues are indicated with gray symbols. The diagonal where both PSIPRED and OSS-HMM *Q*_3_s are equal is shown as a dashed line. OSS-HMM refers to the HMM presented in this article.

We ran the prediction on the 212 sequences, again using single sequences only, with OSS-HMM and PSIPRED. The *per-sequence Q*_3 _scores achieved for each protein chain, by PSIPRED and OSS-HMM, are shown on Figure 3.

EVA dataset includes 20 membrane protein chains : 1pv6:A, 1pw4:A, 1rhz:A, 1zcd:A, 1zhh:B, 2a65:A, 1q90:N, 1q90:L, 1q90:M, 1s51:I, 1s51:J, 1s51:L, 1s51:M, 1s51:T, 1s51:X, 1s51:Z, 1rh5:B, 1rkl:A, 1u4h:A and ls7b:A. As can be seen on Figure 3, PSIPRED achieves good prediction for membrane proteins, whereas the prediction is rather poor with the HMM. Membrane proteins have amino acid propensities very different from those of globular proteins. As membrane proteins were excluded from the learning set to train the HMM, it is not surprising that their HMM-based predictions are largely incorrect. When these 20 sequences are excluded, the global *Q*_3 _score on the remaining 192 proteins is 68.9% for PSIPRED and 68.6% for OSS-HMM, computed on 20 539 residues.

EVA data set also contains very short sequences : 42 sequences are shorter than 50 residues. On very short sequence, a different prediction for a few residues has dramatic consequences on the *Q*_3 _score *per *protein, as shown on Figure 3. Proteins shorter than 50 residues are plotted in gray, they are responsible for the great dispersion of the data. Some rather short sequences are shown in the plot: 1ycy (62 residues), 1r2m (70 residues), 1zeq:X (84 residues), 1zv1A (59 residues), 1r4g:A (53 residues). The mean length of a sequence in the EVA dataset is 189 residues, and 212 residues in the independent test set. The protein length distribution of EVA dataset is shifted toward short sequences when compared to the length distribution in the independent test set (data not shown). In order to analyze the effect of short sequences, and keep sufficient data, a *Q*_3 _score is computed on the "core" of the protein sequences: the five terminal residues of each sequence are excluded from the computation. The global *Q*_3 _score on the "core" of sequences are 68.1% for psipred and 69.2% for OSS-HMM.

This analysis indicates that the performance observed on our independent test set and the EVA dataset both conclude to a better single-sequence based prediction using our OSS-HMM.

#### Confidence scale for the prediction

**Figure 4:**
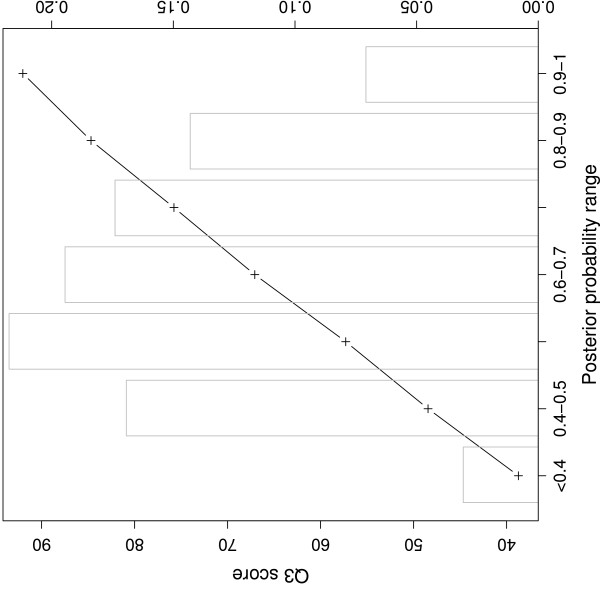
***Q*_3 _score as a function of the posterior probability value**. The Q3 score is computed on the subset of residues that are predicted with probabilities in given ranges. The distribution of residues in the probability ranges is shown as a gray bar-plot. The right axis is related to this distribution.

The posterior probabilities obtained for each structural class can be used as a confidence scale. In Figure 4, we show the *per-residue Q*_3 _scores computed on the independent test set, for residues with posterior probability lying in a given range. As can be seen, the correlation is excellent between posterior probability and the rate of good prediction: residues with high probabilities are better predicted. Thus, the posterior probability given by OSS-HMM is a good indicator of the confidence in the prediction.

#### Influence of the secondary structure assignment software

During the development of our method, several secondary structure assignment methods were tested, both for HMM training and during the prediction evaluation. We tested the STRIDE [42], and KAKSI methods [36]. In Table 4, we report the *per-residue Q*_3 _obtained with three flavors of OSS-HMM trained with KAKSI, DSSP and STRIDE assignments, and PSIPRED, when compared to secondary structure assignments provided by KAKSI, DSSP and STRIDE.

Not surprisingly, a better accuracy is achieved by the HMM when the same assignment method is used for training and evaluation (the estimated standard deviation being 0.2, the *Q*_3 _scores achieved by OSS-HMM_*dssp *_compared to DSSP and STRIDE assignments are equivalent). In these cases, the *Q*_3 _score of OSS-HMM is always higher than the *Q*_3 _score of PSIPRED. It is interesting to note that STRIDE assignments seem to be easier to predict either by OSS-HMM or PSIPRED, even though PSIPRED was trained with DSSP assignments.

#### Comparison with other HMM-based methods

For a precise comparison of OSS-HMM with other HMM-based methods, the evaluation should be done on identical datasets and compared to the same reference assignment. Here, we will only discuss the reported accuracies; conclusion about the possible superiority of a method should be made very cautiously. The first methods that used HMM for protein secondary structure prediction, proposed by Asai et al [9], Stultz et al [10,11], and Goldman et al [12-14] were tested on small datasets. Moreover, the data bank contents are continuously growing, which induces an automatic improvement of the predictive methods because more data are available for training. Thus, the comparison with Asai, Stultz, Goldman and coworkers is not straightforward.

Among recent methods, Brenner et al [17], using a hidden Markov model with a sliding window in secondary structure, reported a *Q*_3 _score of 66.4% on a dataset of 513 sequences, using single sequence only. This result was obtained using the 'CK' mapping of the 8 classes of DSSP into three: (H) = helix, (E) = strand, others = coil, because it yielded a better accuracy than the 'EHL' mapping (see Method section). Using an amino-acid sliding window and amino-acid grouping, Zheng observed a *Q*_3 _score of 67.9% on a small dataset of 76 proteins [18]. Very recently, Aydin et al obtained some very good results with semi-HMM and single sequences: a *Q*_3 _score of 70.3% [22]. Their assignment method was DSSP with the 'CK' mapping and a length correction to remove strands shorter than 3 residues and helices shorter than 5 residues, whereas we use the 'EHL' mapping and no length correction. With the 'EHL' mapping and no length correction, they reported a *Q*_3 _score of 67.4%, which is less than our results with HMM, i. e. 67.9%. To explore the influence of the length correction, we evaluate the *Q*_3 _score on the independent test set of 505 sequences, assigned by DSSP with the length correction. We obtained *Q*_3 _= 69.6%. Thus, it seems that OSS-HMM is, at least as accurate, maybe better than the other methods based on HMM that use single sequences.

#### Prediction using multiple sequences

**Figure 5:**
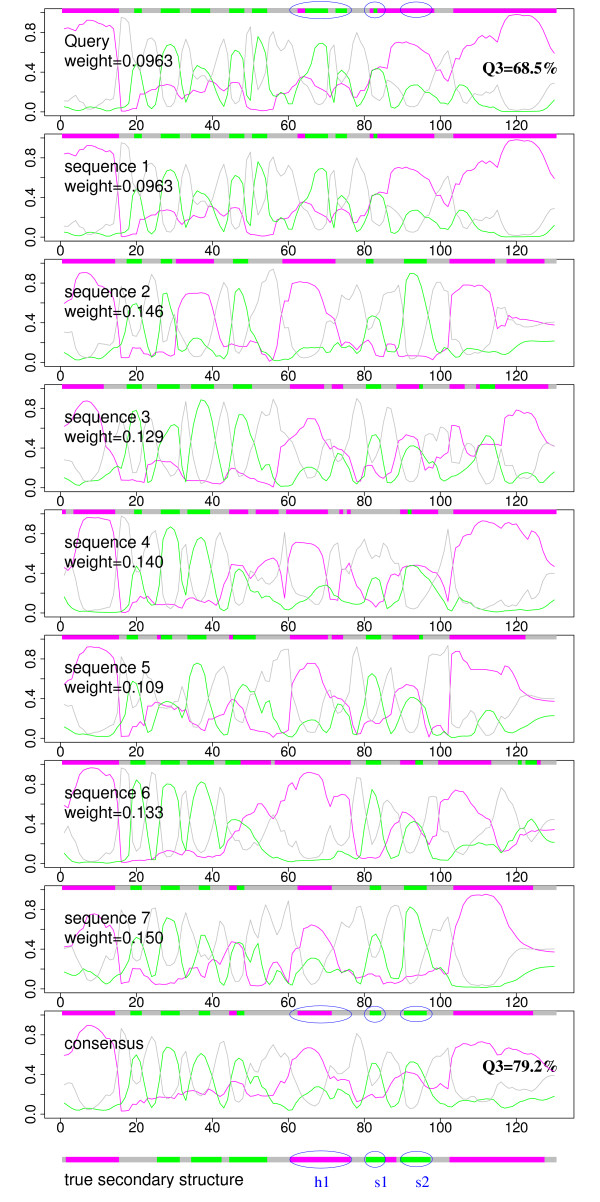
**Example of a multiple sequence prediction for d1jyoa_**. Each plot represents the posterior probabilities of *α*-helix, *β*-strand and coil as a function of the position in the sequence, with the color scheme : magenta = helix, green = strand, grey = coil. "Query" indicates the sequence of the initial sequence d1jyoa_ and "sequence 1" to "sequence 7" are the homologuous sequences retrieved by PSI-BLAST. The Henikoff weight of each sequence is indicated on each plot. "Consensus" indicate the consensus prediction for the sequence family. The predicted secondary structure in each case is shown as a colored bar in the upper part of each plot. The observed secondary structure of d1jyoa_ is plotted in the lower part of the figure. Ellipses focus on zones of the secondary structures that are modified in the prediction using only the query sequence and in the prediction using the multiple sequence alignment.

It is well established that the use of evolutionary information improves the accuracy of secondary structure prediction methods [4,5]. The optimal model OSS-HMM estimated on single sequences can be used to perform the prediction with several sequences without further modification: homologous sequences retrieved by a PSI-BLAST search are independently predicted and the predictions are merged afterward in a consensus prediction. The underlying hypothesis is that homologous sequence share the same secondary structure. An example of multiple sequence based prediction for the SCOP domain d1jyoa_ is shown on Figure 5. Using the sequence of dljyoa_, OSS-HMM achieves a *Q*_3 _score equal to 68.5%. The region around residues 60 to 100, indicated by ellipses, is not correctly predicted. The true secondary structure in this region consists in a *α*-helix of 15 residues (h1), a *β*-strand of 5 residues (b1) followed by a very short helix, and a second *β*-strand of 8 residues (b2) (see segments highlighted by ellipses on Figure 5). Using the single sequence, h1 is predicted as two strand segments and a short helix and s1 and s2 are predicted as one long helix interrupted by one residue in strand. The PSI-BLAST search with the sequence of d1jyoa_ produced a set of 8 sequences. Independent predictions for the 8 sequences show that sequences 2 to 8 have a high probability associated with helix in the region of h1. In the same way, sequences 2, 3 and 7 are predicted correctly as strand in the region s1. Concerning strand s2, only sequences 2 and 7 get a correct prediction. In sequences 3 to 6 this region is predicted as a helix, but have a non negligible probability of strand. The sequence of d1jyoa_ is present twice in the set because it was retrieved by the PSI-BLAST search (sequence 1 on Figure 5). This has no influence on the consensus prediction because the Henikoff weighting scheme ensures that two similar sequences receive the same weight and that the removal of one sequence results in the remaining sequence having a weight that is the double. The Henikoff weights in this sequence family varies from 0.096 to 0.150. Sequences 2 and 7, that contain a correct prediction for s2, have large weights (0.146 and 0.150). The final *Q*_3 _score with the multiple sequences is 79.2%.

Table 5 reports the *per-residue Q*_3 _scores obtained by OSS-HMM trained using various assignment methods, with different assignments taken as reference, on the 516 sequence of the independent test set. PSIPRED is also tested on this data set, using the same data bank for the profile generation. OSS-HMM achieves *Q*_3 _scores of 76.3, 75.5 and 76.7% using respectively KAKSI, DSSP and STRIDE assignments. PSIPRED outperforms OSS-HMM, whatever the assignment used for learning or evaluation with *Q*_3 _scores ranging from 78.6 to 81.2%, depending on the assignment considered as the reference. It should be noted, however, that our HMMs were trained on single sequences and thus may be not optimal for multiple sequence prediction.

The detailed prediction scores of OSS-HMM trained on DSSP assignment and by PSIPRED are reported in Table 6. When compared to the single sequence-based prediction (Table 3), all the prediction scores have increased. *α*-helices are still better predicted than strands and coil: the MCC is equal to 0.7 for helices *vs *0.6 and 0.57 for strands and coil respectively. The sensitivity (*Q*_*obs*_) of *β*-strand prediction is only 56.1%, for a specificity (*Q*_*pred*_) of 80.3%: OSS-HMM predicts too few *β*-strands, but the *β*-strand prediction can be trusted. With single sequence, the sensitivity and specificity of *β*-strands of *β*-strand prediction were respectively equal to 51.9 and 64.5%. The inclusion of homologous sequence information thus greatly increased the specificity and moderately increased the sensitivity of the *β*-strand prediction. Previous studies based on HMM and multiple sequences reported global *Q*_3 _scores of 74.3% by Bystroff et al [16], 72.2% by Brenner et al [17], and 72.8% by Chu et al [21]. Thus, our HMM prediction appears to be more efficient than the other methods reported before, when used with homologous sequences. Moreover, our model is very parsimonious, i. e., has a very limited number of parameters when compared to complex models like the one of Bystroff et al, and neural networks.

In most of the methods that use evolutionary information with hidden Markov models, the likelihood and re-estimation formula are modified to incorporate the probability of a given profile in a hidden state. It is done using various formulation such as multinomial distribution [16,21,43], large deviation [17] or the scalar product of the emission parameters and the observed frequency (with a normalization to obtain a true probability) [44]. In all these cases, the estimation is done on a set of profiles and the input for the prediction is a sequence profile. Another method, more closely related to ours, was proposed by Kall et al [45]. In this approach, the prediction is performed independently for each sequence. The predictions are then used as input for an "optimal accuracy" decoder that ensures that the predicted path is possible within the state graph. This method has the advantage of a satisfactory handling of gaps.

Here, we evaluated the predictive capability of our model, previously trained on single sequences, using a simple voting procedure. Since no strong constraints are applied to the paths within the graphs, we do not need sophisticated algorithm to be in agreement with the model structure. Despite the simplicity of the multiple sequence procedure, the results are very promising: OSS-HMM appears to be as good, maybe more efficient, than previous secondary structure prediction methods based on HMM that use evolutionary information. It would be very interesting to see if the explicit integration of sequence profiles for prediction and estimation as in [16,43,44] could increase the prediction accuracy.

## Conclusion

In this paper we analyzed OSS-HMM, a model generated using an automated design [23], that allows the discovery of new features about the data. This method could in principle be applicable to other similar prediction problems, such as predicting transmembrane helices, splice sites, signal peptides, etc. OSS-HMM has a limited number of parameters, which allows a biological interpretation of the state graph. Mathematically speaking, it is simple: a first order Markov chain for the secondary structure state succession (hidden process) and independent amino-acids, conditionally to the hidden process. Despite its simplicity, it is able to capture some high order correlations such as four-states periodicity in helices and even the specification of distinct types of loops. In this kind of model, high order correlations are implicitly taken into account by the constraints on the transitions. OSS-HMM learned some classical features of secondary structures described previously in other studies: specific sequence signature of helix capping, sequence periodicity of helices and polar/non polar alternation in *β*-strands. New features revealed by our model are the presence of several, well distinct amino acid preferences for *β*-strand capping. To the best of our knowledge, previous studies about *β*-strand specificities did not reveal such strong signatures.

Due to its limited number of parameters, no over-fitting was observed when using OSS-HMM for secondary structure prediction. On single sequences, it performs better than PSIPRED and other HMM-based methods, with a *Q*_3 _score of 67.9%. The use of the same model, OSS-HMM, in a multiple sequence prediction framework gave very promising results with a *Q*_3 _equal to 75.5%. This level of performance is as good, maybe better, than the previous methods based on HMMs and evolutionary information [16,17,21]. The perspectives of this work are twofolds. First, we can re-estimate the model and carry out the prediction using sequence profiles as done in [16,43,44]. A second perspective is the extension to the local structure prediction of non-periodic regions. Most methods predict protein secondary structures as 3 states: helix, strand and coil. Coil is a 'complementary' state for those residues that do neither belong to a helix nor to a strand, accounting for about 50% of all residues. Therefore predicting coils does not provide any information about the residue conformations. One way of extracting more information from the protein sequence is to predict Φ/Ψ zones for coil residues. Such a prediction can be readily included in the HMM and we plan to use it for *de novo *prediction.

## Methods

### Data

The dataset is a subset of 2524 structural domains of ASTRAL 1.65 [46] corresponding to the SCOP domain definition [47]. An initial list of domains with no more than 25% identity within sequence pairs was retrieved on the ASTRAL web page [48]. This list is filtered in order to remove NMR structures, X-ray structures with a resolution factor greater than 2.25 Å, membrane proteins and domains shorter than 50 residues [see Additional file 6].

Secondary structures are assigned by DSSP [49], STRIDE [42] or KAKSI [36]. STRIDE and DSSP assignments are reduced to three classes using the 'EHL' mapping employed in EVA evaluation [3]: (H, G, I) = helix, (E, b) = strand, others (S, T, blank) = coil. The whole dataset contains 492 724 residues with a defined secondary structure.

The dataset is divided into a cross-validation data set and an independent test set containing respectively 2019 sequences (397401 residues) and 505 sequences (95 323 residues). The independent test set is *never *used in parameter estimation and model selection. Models are trained and tested using a four-fold cross-validation procedure on the cross-validation data.

Secondary structure contents are similar in the cross-validation and the independent datasets: 38% helix, 22% strand and 40% coil according to STRIDE assignment and 37% helix, 23% strand and 40% coil according to DSSP assignment.

The method is also tested on a dataset of 212 protein sequences retrieved from the EVA website ("common subset 6" [50]). DSSP secondary structure assignment of the corresponding sequences were also retrieved on this website. This set of 212 protein sequences has been used to evaluate the prediction performance of the methods involved in EVA assessment.

### Hidden Markov models

#### HMM applied to secondary structure prediction

Hidden Markov models are probabilistic models of sequences, appropriate for problems where a sequence is supposed to have hidden features that need to be predicted. The strong theoretical background of HMM (see for example [51]) allows the estimation of model parameters, and the prediction of the hidden features from the observed sequence. The secondary structure prediction task can be expressed in term of a hidden data problem as follows:

• the secondary structure of a particular residue along the sequence is a hidden process,

• the amino acid sequence is the observed process.

The secondary structure succession is governed by a first order Markov chain and amino-acids are independent, conditionally to the hidden process.

In a very basic HMM, each secondary structure is modeled by a single state. The parameters of the model are the transition and emission probabilities. Here, we will consider more complex models with several hidden states *per *secondary structure. The difficulty is to choose the optimal number of hidden states for each secondary structure. Model parameters are then estimated from available data.

#### HMM training

The number of hidden states for each secondary structure and initial parameter values are chosen as explained in the next section. Models are trained with labeled sequence of secondary structures [31] together with the amino-acid sequence using the Expectation-Maximization (EM) algorithm [51]. Secondary structures are labeled according to the DSSP assignment. Labels of secondary structure are removed for the prediction step. All models are trained and handled with the software SHOW [52], extended to handle protein sequences.

#### Optimal HMM selection

Models of increasing size are built without *a priori *knowledge. All transition probabilities are initially set to 1N
 MathType@MTEF@5@5@+=feaafiart1ev1aaatCvAUfKttLearuWrP9MDH5MBPbIqV92AaeXatLxBI9gBaebbnrfifHhDYfgasaacH8akY=wiFfYdH8Gipec8Eeeu0xXdbba9frFj0=OqFfea0dXdd9vqai=hGuQ8kuc9pgc9s8qqaq=dirpe0xb9q8qiLsFr0=vr0=vr0dc8meaabaqaciaacaGaaeqabaqabeGadaaakeaadaWcaaqaaiabigdaXaqaaiabd6eaobaaaaa@2ED1@, *N *being the total number of hidden states. Initial emission parameters are randomly chosen. The similarity of models estimated on different data sets is checked [see Additional file 2]. Ten different starting point are used and the model with the best likelihood is selected during the EM estimation. For large models, 100 starting points are used to verify that the EM estimation has sufficiently explored the parameter space. This procedure provided results similar to those obtained with 10 starting points. The optimal models are then selected using three criteria:

• prediction accuracy assessed by the *Q*_3 _score,

Q3=∑iNiiNtot
 MathType@MTEF@5@5@+=feaafiart1ev1aaatCvAUfKttLearuWrP9MDH5MBPbIqV92AaeXatLxBI9gBaebbnrfifHhDYfgasaacH8akY=wiFfYdH8Gipec8Eeeu0xXdbba9frFj0=OqFfea0dXdd9vqai=hGuQ8kuc9pgc9s8qqaq=dirpe0xb9q8qiLsFr0=vr0=vr0dc8meaabaqaciaacaGaaeqabaqabeGadaaakeaacqWGrbqudaWgaaWcbaGaeG4mamdabeaakiabg2da9maalaaabaWaaabeaeaacqWGobGtdaWgaaWcbaGaemyAaKMaemyAaKgabeaaaeaacqWGPbqAaeqaniabggHiLdaakeaacqWGobGtdaWgaaWcbaGaemiDaqNaem4Ba8MaemiDaqhabeaaaaaaaa@3CF5@

where *N*_*ij *_denotes the number of residues in structure *i *predicted in structure *j*, and *N*_*tot *_the total number of residues,

• Bayesian Information Criterion [53] that ensures the best compromise between the goodness-of-fit of the HMM and a reasonable number of parameters,

*BIC *= *logL *- 0.5 × *k *× *log*(*N*),

where *logL *denotes the log-likelihood of the learning data under the trained model, *k *is the number of independent model parameters and *N *is the size of the training set. *k *is the actual number of free parameters in the final model, i. e., parameters estimated to be null are not counted. The first term of BIC increases with the number of parameters; it is penalized for parsimony by the second term. BIC criterion does not take prediction accuracy into account.

• the statistical distance between two models as described by Rabiner [51]. The symmetric distance *D*_*s *_between two models *M*1 and *M*2 is given by:

Ds(M1,M2)=D(M1,M2)+D(M2,M1)2D(M1,M2)=1T|log⁡L(O(2)|M1)−log⁡L(O(2)|M2)|
 MathType@MTEF@5@5@+=feaafiart1ev1aaatCvAUfKttLearuWrP9MDH5MBPbIqV92AaeXatLxBI9gBaebbnrfifHhDYfgasaacH8akY=wiFfYdH8Gipec8Eeeu0xXdbba9frFj0=OqFfea0dXdd9vqai=hGuQ8kuc9pgc9s8qqaq=dirpe0xb9q8qiLsFr0=vr0=vr0dc8meaabaqaciaacaGaaeqabaqabeGadaaakeaafaqabeGabaaabaGaemiraq0aaSbaaSqaaiabdohaZbqabaGccqGGOaakcqWGnbqtdaWgaaWcbaGaeGymaedabeaakiabcYcaSiabd2eannaaBaaaleaacqaIYaGmaeqaaOGaeiykaKIaeyypa0ZaaSaaaeaacqWGebarcqGGOaakcqWGnbqtcqaIXaqmcqGGSaalcqWGnbqtcqaIYaGmcqGGPaqkcqGHRaWkcqWGebarcqGGOaakcqWGnbqtcqaIYaGmcqGGSaalcqWGnbqtcqaIXaqmcqGGPaqkaeaacqaIYaGmaaaabaGaemiraqKaeiikaGIaemyta0KaeGymaeJaeiilaWIaemyta0KaeGOmaiJaeiykaKIaeyypa0ZaaSaaaeaacqaIXaqmaeaacqWGubavaaGaeiiFaWNagiiBaWMaei4Ba8Maei4zaCMaemitaWKaeiikaGIaem4ta80aaWbaaSqabeaacqGGOaakcqaIYaGmcqGGPaqkaaGccqGG8baFcqWGnbqtdaWgaaWcbaGaeGymaedabeaakiabcMcaPiabgkHiTiGbcYgaSjabc+gaVjabcEgaNjabdYeamjabcIcaOiabd+eapnaaCaaaleqabaGaeiikaGIaeGOmaiJaeiykaKcaaOGaeiiFaWNaemyta00aaSbaaSqaaiabikdaYaqabaGccqGGPaqkcqGG8baFaaaaaa@757E@

where *O*^(2) ^is a sequence of length *T *generated by model *M*_2 _and log *L*(*O*^(2)^|*M*_*i*_) is the log-likelihood of *O*^(2) ^under model *M*_*i*_.

Briefly, the selection is done as follows (see [23] for further details about the selection procedure). Initially, models with equal number of hidden states for each structural class are considered. The three above criteria help selecting models with a limited number of hidden states, about 15 per structural class. Then we consider models for which the number of states increases for one structural class while it remains fixed to one for the two other classes. This defines the model size range that needs to be explored for each structural class: 12 to 16 states for helices, 6 to 10 states for strands and 5 to 13 states for coil. Finally, all 225 models within these size ranges are generated and evaluated. The optimal model, described in the result section, has 36 hidden states: 15 for helices, 9 for strands and 12 for coil. We will refer to this model as OSS-HMM.

#### HMM prediction using single sequences

Predictions are done using the forward/backward algorithm. This allows the computation of the posterior probability of each hidden state in each position of the query sequence, given the model and the whole sequence: *P*(*S*_*t *_= *u *| *X*), where *S*_*t *_is the hidden state at position *t*, *u *is a particular hidden state and *X *is the amino-acid sequence (see, e.g., [54] for computation detail).

The posterior probability of hidden states of the same structural class *c *are summed together:

P(St=s|X)=∑u∈cP(St=u|X),     (1)
 MathType@MTEF@5@5@+=feaafiart1ev1aaatCvAUfKttLearuWrP9MDH5MBPbIqV92AaeXatLxBI9gBaebbnrfifHhDYfgasaacH8akY=wiFfYdH8Gipec8Eeeu0xXdbba9frFj0=OqFfea0dXdd9vqai=hGuQ8kuc9pgc9s8qqaq=dirpe0xb9q8qiLsFr0=vr0=vr0dc8meaabaqaciaacaGaaeqabaqabeGadaaakeaacqWGqbaucqGGOaakcqWGtbWudaWgaaWcbaGaemiDaqhabeaakiabg2da9iabdohaZjabcYha8jabdIfayjabcMcaPiabg2da9maaqafabaGaemiuaaLaeiikaGIaem4uam1aaSbaaSqaaiabdsha0bqabaGccqGH9aqpcqWG1bqDcqGG8baFcqWGybawcqGGPaqkaSqaaiabdwha1jabgIGiolabdogaJbqab0GaeyyeIuoakiabcYcaSiaaxMaacaWLjaWaaeWaaeaacqaIXaqmaiaawIcacaGLPaaaaaa@4E84@

*c *being the set of hidden states modeling the structural class. The structural class with the highest probability is predicted.

#### HMM prediction using multiple sequences

Models estimated with single sequences can be used in a framework of a multiple sequence prediction. The multiple sequence based prediction is carried out in three steps:

1. a PSI-BLAST search is performed using the sequence to be predicted, resulting in a family of *n *homologous sequences,

2. the secondary structures of these *n *sequences are independently predicted using the HMM,

3. the *n *predictions are combined into a unique prediction.

In the first step, homologous sequences are extracted using PSI-BLAST with search parameters -j 3 -h 0.001 -e 0.001, against a non-redundant data bank. The redundancy level of the data bank is 70% (i.e., no pair of sequences has more than 70% identical residues after alignment) and the data bank has been filtered to remove low-complexity regions, transmembrane regions, and coiled-coil segments. Other data banks (80 and 90% redondancy level, filtered or not) were tested and the above choice provided the best results. In the second step, *n *predictions are performed, independently for each sequence retrieved in the first step, with the forward-backward algorithm.

In the third step, the *n *independent predictions are combined. At each position of the sequence family, the predictions of the *n *sequences for the same position contribute to the final prediction, with a sequence weight. Sequence weights are used to correct for the fact that the *n *sequences retrieved in the first step do not necessarily correctly sample the sequence space. For example, a sequence family can be composed of several very similar sequences and a few more distant sequences. Without sequence weights, the final prediction would be biased toward the set of similar sequences and the information carried by the more distant sequences would be lost. The probability of the secondary structure *s *at the position *t *of the sequence family *F *is then given by:

PF(St=s)=∑i=1nθiPi(St=s),     (2)
 MathType@MTEF@5@5@+=feaafiart1ev1aaatCvAUfKttLearuWrP9MDH5MBPbIqV92AaeXatLxBI9gBaebbnrfifHhDYfgasaacH8akY=wiFfYdH8Gipec8Eeeu0xXdbba9frFj0=OqFfea0dXdd9vqai=hGuQ8kuc9pgc9s8qqaq=dirpe0xb9q8qiLsFr0=vr0=vr0dc8meaabaqaciaacaGaaeqabaqabeGadaaakeaacqWGqbaudaWgaaWcbaGaemOrayeabeaakiabcIcaOiabdofatnaaBaaaleaacqWG0baDaeqaaOGaeyypa0Jaem4CamNaeiykaKIaeyypa0ZaaabCaeaaiiGacqWF4oqCdaWgaaWcbaGaemyAaKgabeaakiabdcfaqnaaBaaaleaacqWGPbqAaeqaaOGaeiikaGIaem4uam1aaSbaaSqaaiabdsha0bqabaGccqGH9aqpcqWGZbWCcqGGPaqkaSqaaiabdMgaPjabg2da9iabigdaXaqaaiabd6gaUbqdcqGHris5aOGaeiilaWIaaCzcaiaaxMaadaqadaqaaiabikdaYaGaayjkaiaawMcaaaaa@4FC9@

where *P*_*i *_(*S*_*t *_= *s*) is the posterior probability of the secondary structure *s *obtained for the single sequence *i *at position *t *(see equation 1). The sum is taken over all the *n *sequences retrieved for the family that do not contain gap at the considered position. The secondary structure with the highest probability is taken as the prediction.

Several weighting scheme were tested: Henikoff weights [55], a weighting scheme introduced by Thompson et al [56] that uses phylogenetic trees, and a new weighting scheme based on information sharing in phylogenetic trees [see Additional file 4].

We also developed a more sophisticated multiple sequence prediction in which the HMM is fed with the sequence families and the corresponding phylogenetic trees [see Additional file 4]. In this case, it is supposed that aligned sequences not only share the same secondary structure but the same path of hidden states.

The best results were obtained using Henikoff weighting scheme and are presented in the Result section.

### Assessing the prediction

Accuracy of secondary structure prediction is assessed using standard scores such as *Q*_3_, SOV [57], *Q*_*obs *_(sensitivity), *Q*_*pred *_(specificity) and Matthew's correlation coefficient [see Additional file 1].

## Availability and requirements

Project name:

OSS-EMM

Project home page:



Operating system:

Linux

Programming language:

C++, perl, python.

Other requirements:

GSL library (1.3 or higher, ).

Licence:

GNU GPL

Any restrictions to use by non-academics:

no.

## Authors' contributions

JM conceived the models and carried out the analysis. JM, JFG and FR conceived the study and participated in its design and coordination. All authors read and approved the final manuscript.

## Supplementary Material

Additional file 1 **Definition of prediction scores**. Definitions of *Q*_3_, *Q*_*obs*_, *Q*_*pred*_, MCC and SOV scores. Click here for file

Additional file 2 **Comparing hidden Markov models**. Elements about the problem of comparing HMMs to ensure that the model is interpretable. Click here for file

Additional file 3 **Simulation study**. Analysis of amino-acid composition of sequences that are simulated with OSS-HMM and a comparison to real data. Click here for file

Additional file 4 **Handling homologs with HMM**. Overview of other approaches we developed to integrate evolutionary information in the prediction. Click here for file

Additional file 5 **Classification of protein sequences using paths in the HMM**. Attempt to classify protein sequences using their paths in OSS-HMM. Click here for file

Additional file 6 **URL to retrieve the domain lists**. Url to retrieve the cross-validation and test data sets. Click here for file

## Figures and Tables

**Table 1:** Number of transitions in the final 36 states HMM

Transitions	*N*_*init*_	*N*_*final*_	*N*_>0.1_	Ratio_*final*_
Helix to helix	225	81	24	36%
Helix to coil	180	47	9	26%
Helix to strand	135	21	0	15%

Strand to Strand	81	46	20	57%
Strand to coil	108	50	5	46%
Strand to helix	135	17	0	13%

Coil to coil	144	98	25	68%
Coil to helix	180	31	2	17%
Coil to strand	108	57	4	29%

Total	1296	448	89	34%

*N*_*init*_: number of allowed transitions in the initial model, *N*_*final*_: number of non-null transitions in the final model, *N*_>0.1_: number of transitions associated with probabilities greater than 0.1 in the final model, Ratio_*final*_: percentage of non-null transitions relative to the initial number.

**Table 2:** Occurences of coil states in different types of loops in the Viterbi paths of the cross-validation data set

	*α*/*α*	*β*/*β*	*α*/*β*	*β*/*α*	frequency
c1	3308	57	30	3962	5.2
c2	2686	10374	2920	6941	16.1
c3	14	5880	124	3014	6.3
c4	1953	151	2312	170	3.2
c5	2327	225	3101	231	4.1
c6	5276	2894	5609	2579	11.5
c7	3165	6684	2996	5691	13.0
c8	1677	6364	3054	2223	9.4
c9	2	2382	429	7	2.0
c10	1359	6670	3375	1671	9.2
c11	2828	5477	3585	2748	10.3
c12	3969	2943	4882	2025	9.7
frequency	20.1	35.2	22.8	21.9	

Frequency denotes the marginal distribution of each variable (hidden states or loop type), in percentage.

**Table 3:** Prediction accuracy obtained for the 505 sequences of the independent test set, using single sequences

	OSS-HMM				PSIPRED			
	*Q*_*obs*_	*Q*_*pred*_	MCC	SOV	*Q*_*obs*_	*Q*_*pred*_	MCC	SOV
Helix	72.1	72.5	0.56	67.7	70.5	70.4	0.53	66.7
Strand	51.9	64.5	0.47	58.3	56.6	60.8	0.47	60.5
Coil	73.2	65.5	0.47	63.9	69.2	66.7	0.46	61.9

	*Q*_3_	SOV			*Q*_3_	SOV		

Global	67.9	63.9			66.8	63.3		

OSS-HMM refers to the HMM with 36 hidden states presented in this article.

**Table 4:** Influence of the assignment method upon the *Q*_3 _score obtained for the 505 sequences of the independent test set

	Testkaksia MathType@MTEF@5@5@+=feaafiart1ev1aaatCvAUfKttLearuWrP9MDH5MBPbIqV92AaeXatLxBI9gBaebbnrfifHhDYfgasaacH8akY=wiFfYdH8Gipec8Eeeu0xXdbba9frFj0=OqFfea0dXdd9vqai=hGuQ8kuc9pgc9s8qqaq=dirpe0xb9q8qiLsFr0=vr0=vr0dc8meaabaqaciaacaGaaeqabaqabeGadaaakeaacqqGubavcqqGLbqzcqqGZbWCcqqG0baDdaqhaaWcbaGaem4AaSMaemyyaeMaem4AaSMaem4CamNaemyAaKgabaGaemyyaegaaaaa@3A53@	Testdsspb MathType@MTEF@5@5@+=feaafiart1ev1aaatCvAUfKttLearuWrP9MDH5MBPbIqV92AaeXatLxBI9gBaebbnrfifHhDYfgasaacH8akY=wiFfYdH8Gipec8Eeeu0xXdbba9frFj0=OqFfea0dXdd9vqai=hGuQ8kuc9pgc9s8qqaq=dirpe0xb9q8qiLsFr0=vr0=vr0dc8meaabaqaciaacaGaaeqabaqabeGadaaakeaacqqGubavcqqGLbqzcqqGZbWCcqqG0baDdaqhaaWcbaGaemizaqMaem4CamNaem4CamNaemiCaahabaGaemOyaigaaaaa@391A@	Teststridec MathType@MTEF@5@5@+=feaafiart1ev1aaatCvAUfKttLearuWrP9MDH5MBPbIqV92AaeXatLxBI9gBaebbnrfifHhDYfgasaacH8akY=wiFfYdH8Gipec8Eeeu0xXdbba9frFj0=OqFfea0dXdd9vqai=hGuQ8kuc9pgc9s8qqaq=dirpe0xb9q8qiLsFr0=vr0=vr0dc8meaabaqaciaacaGaaeqabaqabeGadaaakeaacqqGubavcqqGLbqzcqqGZbWCcqqG0baDdaqhaaWcbaGaem4CamNaemiDaqNaemOCaiNaemyAaKMaemizaqMaemyzaugabaGaem4yamgaaaaa@3BD0@
OSS-HMMkaksid MathType@MTEF@5@5@+=feaafiart1ev1aaatCvAUfKttLearuWrP9MDH5MBPbIqV92AaeXatLxBI9gBaebbnrfifHhDYfgasaacH8akY=wiFfYdH8Gipec8Eeeu0xXdbba9frFj0=OqFfea0dXdd9vqai=hGuQ8kuc9pgc9s8qqaq=dirpe0xb9q8qiLsFr0=vr0=vr0dc8meaabaqaciaacaGaaeqabaqabeGadaaakeaacqqGpbWtcqqGtbWucqqGtbWucqqGTaqlcqqGibascqqGnbqtcqqGnbqtdaqhaaWcbaGaem4AaSMaemyyaeMaem4AaSMaem4CamNaemyAaKgabaGaemizaqgaaaaa@3CB6@	**68.1**	67.5	66.4
OSS-HMM_*dssp*_	66.5	**67.9**	**68.3**
OSS-HMM_*stride*_	67.1	67.8	**68.8**
PSIPRED	65.7	66.7	**67.0**

^*a*^: evaluation using KAKSI assignment, ^*b*^: evaluation using DSSP assignment, ^*c*^: evaluation using STRIDE assignment. ^*d*^: OSS-HMM_*x *_denotes 36 state HMM trained using *x *assignment.

**Table 5:** *Q*_3 _scores obtained for the 505 sequences of the independent test set using multiple sequence information

	Testkaksia MathType@MTEF@5@5@+=feaafiart1ev1aaatCvAUfKttLearuWrP9MDH5MBPbIqV92AaeXatLxBI9gBaebbnrfifHhDYfgasaacH8akY=wiFfYdH8Gipec8Eeeu0xXdbba9frFj0=OqFfea0dXdd9vqai=hGuQ8kuc9pgc9s8qqaq=dirpe0xb9q8qiLsFr0=vr0=vr0dc8meaabaqaciaacaGaaeqabaqabeGadaaakeaacqqGubavcqqGLbqzcqqGZbWCcqqG0baDdaqhaaWcbaGaem4AaSMaemyyaeMaem4AaSMaem4CamNaemyAaKgabaGaemyyaegaaaaa@3A53@	Testdsspb MathType@MTEF@5@5@+=feaafiart1ev1aaatCvAUfKttLearuWrP9MDH5MBPbIqV92AaeXatLxBI9gBaebbnrfifHhDYfgasaacH8akY=wiFfYdH8Gipec8Eeeu0xXdbba9frFj0=OqFfea0dXdd9vqai=hGuQ8kuc9pgc9s8qqaq=dirpe0xb9q8qiLsFr0=vr0=vr0dc8meaabaqaciaacaGaaeqabaqabeGadaaakeaacqqGubavcqqGLbqzcqqGZbWCcqqG0baDdaqhaaWcbaGaemizaqMaem4CamNaem4CamNaemiCaahabaGaemOyaigaaaaa@391A@	Teststridec MathType@MTEF@5@5@+=feaafiart1ev1aaatCvAUfKttLearuWrP9MDH5MBPbIqV92AaeXatLxBI9gBaebbnrfifHhDYfgasaacH8akY=wiFfYdH8Gipec8Eeeu0xXdbba9frFj0=OqFfea0dXdd9vqai=hGuQ8kuc9pgc9s8qqaq=dirpe0xb9q8qiLsFr0=vr0=vr0dc8meaabaqaciaacaGaaeqabaqabeGadaaakeaacqqGubavcqqGLbqzcqqGZbWCcqqG0baDdaqhaaWcbaGaem4CamNaemiDaqNaemOCaiNaemyAaKMaemizaqMaemyzaugabaGaem4yamgaaaaa@3BD0@
OSS-HMMkaksid MathType@MTEF@5@5@+=feaafiart1ev1aaatCvAUfKttLearuWrP9MDH5MBPbIqV92AaeXatLxBI9gBaebbnrfifHhDYfgasaacH8akY=wiFfYdH8Gipec8Eeeu0xXdbba9frFj0=OqFfea0dXdd9vqai=hGuQ8kuc9pgc9s8qqaq=dirpe0xb9q8qiLsFr0=vr0=vr0dc8meaabaqaciaacaGaaeqabaqabeGadaaakeaacqqGpbWtcqqGtbWucqqGtbWucqqGTaqlcqqGibascqqGnbqtcqqGnbqtdaqhaaWcbaGaem4AaSMaemyyaeMaem4AaSMaem4CamNaemyAaKgabaGaemizaqgaaaaa@3CB6@	76.3	74.4	75.2
OSS-HMM_*dssp*_	73.8	75.5	75.4
OSS-HMM_*stride*_	75.3	76.02	76.7
PSIPRED	78.6	80.5	81.2

^*a*^: evaluation using kaksi assignment, ^*b*^: evaluation using DSSP assignment, ^*c*^: evaluation using STRIDE assignment. ^*d*^: OSS-HMM_*x *_denotes 36 state HMM trained using *x *assignment.

**Table 6:** Prediction scores obtained for the 505 sequences of the independent test set using multiple sequence information

	OSS-HMMdsspa MathType@MTEF@5@5@+=feaafiart1ev1aaatCvAUfKttLearuWrP9MDH5MBPbIqV92AaeXatLxBI9gBaebbnrfifHhDYfgasaacH8akY=wiFfYdH8Gipec8Eeeu0xXdbba9frFj0=OqFfea0dXdd9vqai=hGuQ8kuc9pgc9s8qqaq=dirpe0xb9q8qiLsFr0=vr0=vr0dc8meaabaqaciaacaGaaeqabaqabeGadaaakeaacqqGpbWtcqqGtbWucqqGtbWucqqGTaqlcqqGibascqqGnbqtcqqGnbqtdaqhaaWcbaGaemizaqMaem4CamNaem4CamNaemiCaahabaGaemyyaegaaaaa@3B75@				PSIPRED			
	Qobsb MathType@MTEF@5@5@+=feaafiart1ev1aaatCvAUfKttLearuWrP9MDH5MBPbIqV92AaeXatLxBI9gBaebbnrfifHhDYfgasaacH8akY=wiFfYdH8Gipec8Eeeu0xXdbba9frFj0=OqFfea0dXdd9vqai=hGuQ8kuc9pgc9s8qqaq=dirpe0xb9q8qiLsFr0=vr0=vr0dc8meaabaqaciaacaGaaeqabaqabeGadaaakeaacqWGrbqudaqhaaWcbaGaem4Ba8MaemOyaiMaem4CamhabaGaemOyaigaaaaa@3374@	*Q*_*pred*_	MCC	SOV	*Q*_*obs*_	*Q*_*pred*_	MCC	SOV
Helix	78.5	83.3	0.70	77.5	85.3	85.9	0.77	85.0
Strand	56.1	80.3	0.60	65.1	76.1	78.2	0.70	78.9
Coil	83.8	68.4	0.57	70.8	78.6	76.9	0.63	71.5

	*Q*_3_	SOV			*Q*_3_			

Global	75.5	72.0			80.5	78.6		

^*a*^: OSS-HMM_*dssp *_denotes 36 state HMM trained using dssp assignment, ^*b*^: predictions scores are reported with the dssp assignment taken as the reference.
