# Supplementary material for: Analysis of an optimal hidden Markov model for secondary structure prediction
Source: BMC Struct Biol. 2006 Dec 13;6:25. doi: 10.1186/1472-6807-6-25 (PMC1769381; doi:10.1186/1472-6807-6-25)
Supplement: Additional file 1 — Definition of prediction scores. Definitions of Q3, Qobs, Qpred, MCC and SOV scores. [file 1472-6807-6-25-S1.pdf]

# Additional file 1 - Definition of prediction scores

The following scores are used to evaluate the prediction:

- $Q_3$ score. The $Q_3$ score can be computed on a *per-residue* basis, i.e., globally for a set of sequences, or on a *per-sequence* basis, i.e. separately for each sequence.

- Segment Overlapping score (SOV). It is defined, for the secondary structure $i$, by:

$$SOV(i) = \frac{1}{N(i)} \sum_{s(i)} \frac{minov(s_1, s_2) + delta(s_1, s_2)}{maxov(s_1, s_2)} \times len(s_1)$$

  with the normalization value $N(i)$ defined as:

$$N(i) = \sum_{s(i)} len(s_1) + \sum_{S'(i)} len(s_1).$$

  $s_1$ refers to the secondary structure assignment and $s_2$ to the prediction. The sums on $s(i)$ are taken over all the segment pairs in state $i$ which overlap by at least one residue. The sum on $s'(i)$ is taken over the remaining segments in state $i$ found in the assignment, $len(s_1)$ is the number of residues in segment $s_1$, $minov(s_1, s_2)$ is the length of overlap of $s_1$ and $s_2$ (intersection of $s_1$ and $s_2$), $maxov(s_1, s_2)$ is the total extend for which at least one of the segments $s_1$ and $s_2$ has a residue in state $i$ (union of $s_1$ and $s_2$), and $delta(s_1, s_2)$ is defined as:

$$min\left\{maxov(s_1, s_2) - minov(s_1, s_2); minov(s_1, s_2); int(len(s_1)/2); int(len(s_2)/2)\right\},$$

  where $min\{x1; x2; x3; ...; xn\}$ is the minimum of $n$ integers. SOV varies from 0 to 100%. The higher the SOV value, the better is the agreement between real and secondary structure, in terms of segments.

- $Q_{obs}(i)$, or sensitivity, for the structural class $i$. It is defined as:

$$Q_{obs}(i) = \frac{N_{ii}}{N_{obs}(i)},$$

  where $N_{ii}$ denotes the number of residues in structure $i$ predicted in structure $i$ and $N_{obs}(i)$ the number of residues observed in structure $i$,

- $Q_{pred}(i)$, or specificity, for the structural class $i$. It is defined as:

$$Q_{pred}(i) = \frac{N_{ii}}{N_{pred}(i)},$$

  where $N_{pred}(i)$ denotes the number of residues predicted in structure i,

- Matthew's correlation coefficient. It is defined by:

$$MCC_i = \frac{tp \times tn - fp \times fn}{\sqrt{(tp + fn)(tp + fp)(tn + fn)(tn + fp)}},$$

  where $tp$, $fp$, $tn$ and $fn$ denote respectively the number of true positive, false positive, true negative and false negative prediction for the structure $i$. $MCC$ is 1 for a perfect prediction and 0 for a random prediction.
