# Supplementary material for: Analysis of an optimal hidden Markov model for secondary structure prediction
Source: BMC Struct Biol. 2006 Dec 13;6:25. doi: 10.1186/1472-6807-6-25 (PMC1769381; doi:10.1186/1472-6807-6-25)
Supplement: Additional file 2 — Comparing hidden Markov models. Elements about the problem of comparing HMMs to ensure that the model is interpretable. [file 1472-6807-6-25-S2.pdf]

Additional file 2 - Comparing hidden Markov models

During this work, we generated many hidden Markov models with random emission parameters and uninformative transition probabilities. A large number of starting points were considered to ensure that the model did not get trapped into local maxima. As we considered only models with a relatively small number of states and obtained no over-fitting, we hypothesized, the model is not over-parametrized. This is a necessary condition for the model to be interpretable. In that case, models obtained from different starting points ought to be similar. In our study, we used a four-fold cross-validation procedure to train and test our models. Here, we explain how we ensure that two models A and B, trained on two different cross-validation partitions (1518 sequences) are similar. It should be noted that some minor differences are expected because these models are trained on datasets that share only 30% of the sequences.

Let us consider two models, A and B, both, say, with 15 states for helices, 12 for coil and 9 for strands. To evaluate their similarity, we need to identify pairs of similar states in the two models. Similar states in the models are characterized by similar emission parameters and transition probabilities. To compare two models we applied the following method:

This can be done by comparing the state emission parameters. The comparison of transition parameters, however, need the previous identification of state pairs. For this reason, the comparison of two models is not a basic task. We applied the following method:

1. For each state of model A, identify the most similar state in model B. The similarity between two states is measured by the Manhattan distance between the emission laws. Only comparison between pairs of states modeling the same secondary structure are allowed.

2. The same procedure, for each state of model B, permits the identification of the most similar state in model A.

3. "exact pairs" are defined as the pairs that are found both ways in the previous two steps (this is similar, in essence, to the *reciprocal best hits* that is used to identify orthologous sequences).

4. For the remaining states, try to assign "extended pairs", defined as pairs of states with low Manhattan distances between their emission laws,

5. Check that the global model topology is conserved (not measured) when state pairs are considered.

The 4-fold cross-validation generates 4 models having 36 hidden states. Six comparisons can be carried out between these models. During these comparisons we obtained an average number of 25 "exact" pairs. This figure increases to 32 pairs if we include the "extended pairs". These results led us to judge that the topology of the different models generated by the cross-validation was similar.
