# Supplementary material for: Analysis of an optimal hidden Markov model for secondary structure prediction
Source: BMC Struct Biol. 2006 Dec 13;6:25. doi: 10.1186/1472-6807-6-25 (PMC1769381; doi:10.1186/1472-6807-6-25)
Supplement: Additional file 3 — Simulation study. Analysis of amino-acid composition of sequences that are simulated with OSS-HMM and a comparison to real data. [file 1472-6807-6-25-S3.pdf]

Additional file 3 - Sequence simulation study

A sequence simulation study was carried out using OSS-HMM presented here. A dataset of 2 019 sequences was generated, with sequence lengths identical to the sequence lengths of the real dataset used in the study. This resulted in a simulated dataset of 398 215 residues.

The secondary structure content of the simulated and real data sets are shown in Table 1.

	simu	real
α	37.1	37.0
β	22.2	22.7
coil	40.7	40.3
total	100%	100%

Table 1: Secondary structure composition of the simulated and real datasets: *simu* and *real* refer respectively to the simulated and real datasets. Percentages of the 3 secondary structures in each data sets are reported. Secondary structures of the real dataset are assigned with DSSP.

The amino-acid compositions of helix, strand and coil segments for the simulated and real data sets are shown in Tables 2. Compositions are similar in both data sets, as assessed by Chi Square tests: p-values are, respectively, 0.92, 0.96 and 0.94 for helices, strands and coil.

	Helices		Strands		Coils	
	simu	real	simu	real	simu	real
A	11.61	11.72	6.14	6.25	6.65	6.62
C	1.17	1.15	1.74	1.72	1.30	1.29
D	5.13	5.17	3.37	3.33	8.11	8.05
E	9.02	9.18	4.56	4.47	5.63	5.56
F	3.93	3.89	5.53	5.47	3.07	3.09
G	3.61	3.58	4.93	4.96	12.45	12.49
H	2.10	2.08	2.38	2.29	2.52	2.53
I	5.64	5.58	10.11	10.08	3.32	3.32
K	6.56	6.70	4.75	4.70	5.57	5.67
L	11.67	11.65	10.01	10.13	6.03	6.12
M	2.53	2.48	2.15	2.09	1.56	1.63
N	3.31	3.31	2.73	2.74	6.17	6.16
P	2.59	2.55	2.03	2.07	8.11	8.11
Q	4.78	4.78	2.89	2.91	3.22	3.26
R	6.09	6.00	4.52	4.52	4.51	4.41
S	4.82	4.80	4.90	5.00	7.32	7.24
T	4.16	4.20	6.75	6.82	6.19	6.12
V	6.23	6.18	13.51	13.58	4.42	4.43
W	1.57	1.52	1.87	1.80	1.05	1.06
Y	3.50	3.47	5.13	5.04	2.83	2.80

Table 2: Amino-acid composition of the simulated and real datasets: *simu* and *real* refer respectively to the simulated of real datasets. Percentages of the 20 amino-acids in each data sets, for each secondary structure, are reported.

The real dataset is composed of 15 661 α-helices, 20 381 β-strands and 36 479 coil segments for a total of 397 401 residues. The simulated dataset is composed of 18 443 α-helices, 21 639 β-strands and 36 889 coil segments for a total of 398 215 residues. The number of helices and strands is slightly greater in the simulated dataset.

We further analyze the length distribution of secondary structure elements in real and simulated datasets.

The resulting distributions are shown on Figure 1. The simulated dataset contains many very short helices (helices of 1-2 residues) that do not occur in the real dataset. Indeed, no constraint is set in the HMM to prevent the simulation of very short segments. Except for very short segments, the length

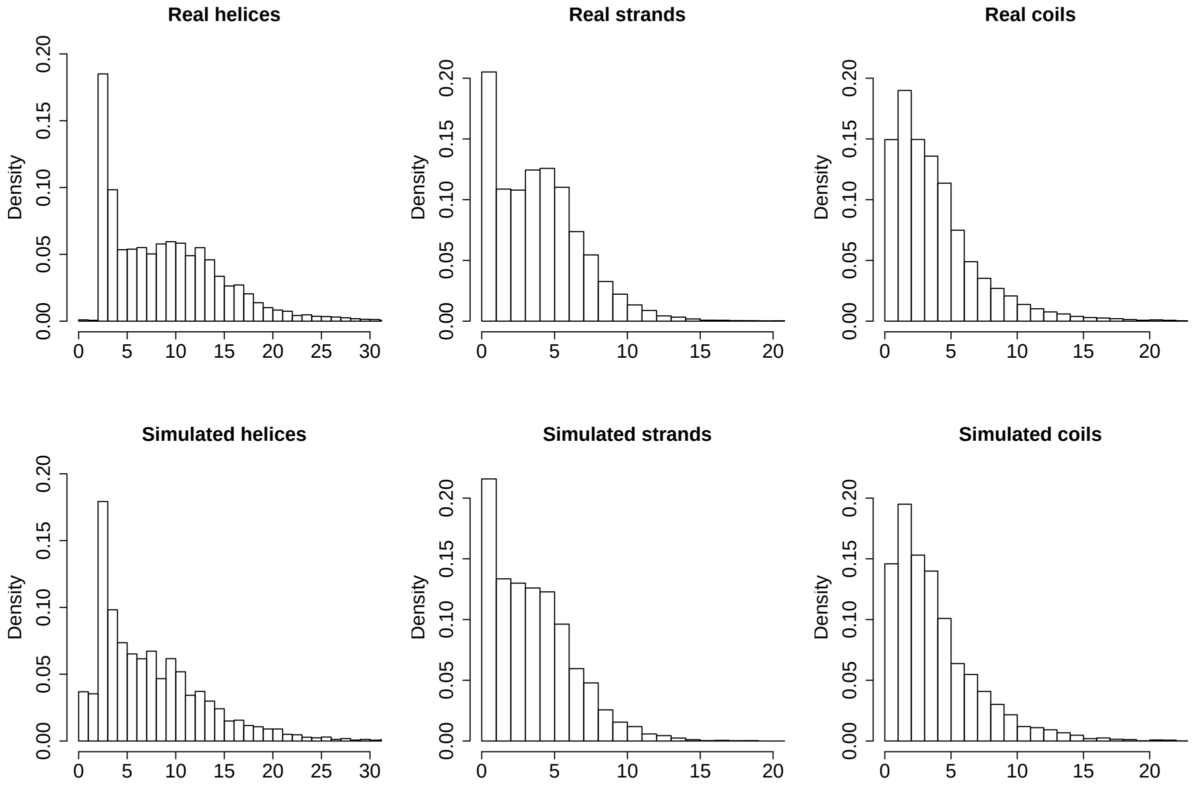

Figure 1: Length distribution of secondary structure segments in real protein structures and sequences simulated by OSS-HMM. The secondary structure of the real dataset is assigned by STRIDE.

distribution of real and simulated helices and strands are similar. Length distributions of coil segments are similar in real and simulated datasets.
