# Supplementary material for: Analysis of an optimal hidden Markov model for secondary structure prediction
Source: BMC Struct Biol. 2006 Dec 13;6:25. doi: 10.1186/1472-6807-6-25 (PMC1769381; doi:10.1186/1472-6807-6-25)
Supplement: Additional file 4 — Handling homologs with HMM. Overview of other approaches we developed to integrate evolutionary information in the prediction. [file 1472-6807-6-25-S4.pdf]

Additional file 4 - Handling homologs with HMM

We present the different strategies we developed to take into account evolutionary information in the secondary structure prediction with the optimal HMM. This includes two kinds of approach:

1. the combination, using different the weighting schemes, of independent predictions performed with the HMM,

2. the coupling of a HMM with a phylogenetic tree.

The later approach is more involved than the straightforward combination of predictions but, unfortunately, provides worst results. We checked the validity of this approach on simulated sequences. This analysis showed that the poor performance on real data is likely due to the violation of the central hypothesis on which this approach rests, namely that hidden states within the HMM are conserved across protein families.

1 Combining independent predictions

In the paper, we present results obtained with the Henikoff weighting scheme [2]. Other weighting schemes have been tested. We give here the definition of these weighting schemes and the results we obtained with them.

1.1 Henikoff weights [2]

Given a multiple sequence alignment (MSA), the weight associated to sequence i at position p of the alignment is given by:

$$w_i^t = \frac{1}{n_{diff}^t \times n_{x_i}^t}$$

where n_{diff}^t is the number of different amino-acids observed at position t of the MSA and $n_{x_i}^t$, is the frequency, at position p of the MSA, of the amino-acid present in sequence i. The final Henikoff weight of sequence i is the mean of positional weights.

1.2 Thompson weights [4]

Let consider the rooted phylogenetic tree shown in Figure 1, where l_j is the length of the edge upstream node j, and O_j is the number of sequences in the subtree downstream the node j.

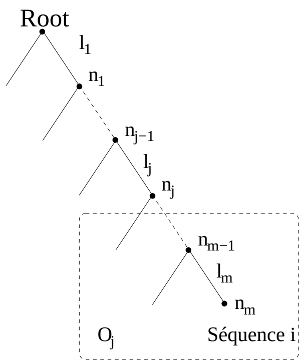

Figure 1: Schematic representation of a rooted tree

The weight of sequence i is given by:

$$w_i = \sum_{j=1}^{m} \frac{l_j}{O_j}.$$

Weights are normalized to sum to 1.

Unrooted phylogenetic trees are obtained from the MSA using the Neighbor Joining algorithm using Kimura distances with the GCG package. Edge length is expressed as mutation number for 100 residues. Unrooted trees are then rooted using a mid-point method at a position where the means of the edge lengths on either side of the root are equal [4].

1.3 Equitable sharing weights

We propose a new weighting scheme based on phylogenetic trees. The idea is to estimate the *real* frequency of amino-acids: how many times a given amino-acid appears independently in a phylogenetic tree. Sequence weights are then derived from their locations in the tree.

Unrooted phylogenetic trees are obtained from the MSA with the Neighbor Joining algorithm of the GCG package that makes use of Kimura distances. The positioning of the root has no influence on further computations because we suppose the the evolution model is reversible and the root does not play any particular role in the following formulas.

Let us consider the phylogenetic trees shown on Figure 2. Trees 1 and 2 both have 7 sequences, two of them with letter a and five of them with letter b. The *real* occurrence of a in tree 1 is probably 1 because the sequences with letter a are separated by only one node. In tree 2, sequences 1 and 6, that bear a, are separated by more nodes. The fact that all other sequences have a b indicates that the *real* frequency of a is probably 2. Trees 3 and 4 illustrate the concept of equitable sharing. In tree 3, sequences with letters a are in the same region of tree and thus receive equivalent weights. On the contrary, in tree 4, sequence 6 is found isolated from other sequences with an a and will then have a larger weight than sequences 1 and 2.

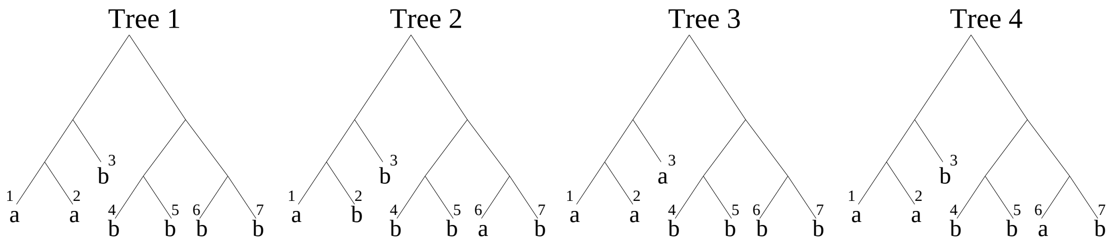

Figure 2: Illustrative phylogenetic trees.

1.3.1 Notations

R is the root of the tree.
g indicates a node.
A terminal node is a leaf.
g_0 and g_1 are the sons of g
A_g is the subtree rooted by g and A is the full tree (see Figure 3)
Two sequences are identical by descent if no mutation occur in the path between them.
$g_0 \equiv g_1$ means that g_0 and g_1 are identical by descent.
$g_0 \equiv A_{g_0}$ is the event: A_{g_0} contains a sequence that is identical by descent to g_0.
$A_{g_0} \equiv A_{g_1}$ is the event: one sequence of A_{g_0} is identical by descent to one sequence of A_{g_1}.
Φ is the set of leafs and Φ_g is the set of leafs of A_g.

Positions in a MSA are considered independents. Formula are presented here for one position. The final weights are the average of positional weights.

The computation of sequence weights requires the previous computation of *real frequencies* of amino-acids in the tree.

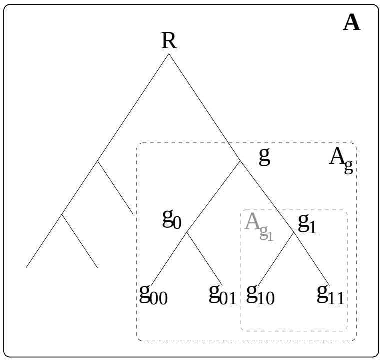

Figure 3:

1.3.2 Computation of *real frequencies*

The *real frequency* of letter a in the tree,$I_a(A \mid \Phi)$, is given by:

$$I_a(A \mid \Phi) = n(a) - \sum_{g \in G} P[g_o \equiv A_{g_0}, g_1 \equiv Ag_1, g_0 \equiv g_1, g = a \mid \Phi] \quad (1)$$

with $n(a)$ being the observed frequency of a and G the set of internal nodes, including the root.

If the evolution process is reversible, expected real frequencies equal, for each amino-acid, the expected of high IBD (identical by descent) classes.

Justification of equation 1 Consider a tree A made of subtrees A_0 and A_1. The real frequency of a in A is the sum of *real frequencies* in A_0 and A_1, corrected by the probability that one sequence in A_0 is identical by descent to one sequence in A_1.

$$I_a(A \mid \Phi) = I_a(A_0 \mid \Phi) + I_a(A_1 \mid \Phi) - P[g \equiv A_0, g \equiv A_1, g_0 \equiv g_1, g = a \mid \Phi]$$

One sequence in A_0 identical by descent to one in A_1 implies that g_0 and g_1 are identical by descent to their common parent g. In the same way, for A_0 and A_1:

$$I_a(A_0 \mid \Phi) = I_a(A_{00} \mid \Phi) + I_a(A_{01} \mid \Phi) - P[g_0 \equiv A_{00}, g_0 \equiv A_{01}, g_{00} \equiv g_{01}, g_0 = a \mid \Phi]$$

$$I_a(A_1 \mid \Phi) = I_a(A_{10} \mid \Phi) + I_a(A_{11} \mid \Phi) - P[g_1 \equiv A_{10}, g_1 \equiv A_{11}, g_{10} \equiv g_{11}, g_1 = a \mid \Phi]$$

The development for all nodes lead to equation 1.

Let us note:

$$Q_a^{g_0} = P(g_0 \equiv A_{g_0} \mid g_0 = a, \Phi_{g_0})$$
$$P_a^{g_0} = P(g_0 \equiv g \mid g = a, \Phi_{g_0})$$
$$\Pi_a^g = P(g = a \mid \Phi)$$

We then obtain:

$$I_a = n(a) - \sum_{g \in G} Q_a^{g_0} \times P_a^{g_0} \times Q_a^{g_1} \times P_a^{g_1} \times \Pi_a^g$$

Computation of $Q_a^g = P(g \equiv A_g \mid g = a, \Phi_g)$

$$\begin{cases} Q_a^g = 1_{\{g=a\}} & \text{if g is a leaf} \\ Q_a^g = 1 - (1 - P_a^{g_0} Q_a^{g_0})(1 - P_a^{g_1} Q_a^{g_1}) & \text{if g is an internal node.} \end{cases}$$

Computation of $P_a^{g_0} = P(g_0 \equiv g \mid g = a, \Phi_{g_0})$

$$
\begin{aligned}
P_a^{g_0} &= \frac{P(g_0 \equiv g \mid g = a) P(\Phi_{g_0} \mid g = a, g_0 \equiv g)}{P(\Phi_{g_0} \mid g = a)} \quad \text{Bayes theorem} \\
&= \frac{P(g_0 \equiv g \mid g = a) P(\Phi_{g_0} \mid g_0 = a)}{P(\Phi_{g_0} \mid g = a)} \\
&= \frac{P(g_0 \equiv g \mid g = a) P(\Phi_{g_0} \mid g_0 = a)}{\sum_b P(\Phi_{g_0} \mid g_0 = b) P(g_0 = b \mid g = a)} \\
&= \frac{F_a^{g_0} P(g_0 \equiv g = a)}{\sum_b F_b^{g_0} P(g_0 = b \mid g = a)} \quad \text{if we note } F_a^g = P(\Phi_g \mid g = a)
\end{aligned}
$$

$P(g_0 \equiv g) = 1 - l$ with l, the length of the edge from g to g_0.

$$
\begin{cases}
P(g_0 = b \mid g = a) = 1 - l + l\pi(b) & \text{if a=b} \\
P(g_0 = b \mid g = a) = l\pi(b) & \text{else}
\end{cases}
$$

where π denotes the renewal probability, here, the relative frequency of amino-acids in the database.

Computation of $F_a^g = P(\Phi_g \mid g = a)$

$$
\begin{aligned}
F_a^g &= 1_{\{g=a\}} \text{ if g is a leaf} \\
F_a^g &= [P(g \equiv g_0) F_a^{g0} + (1 - P(g \equiv g_0)) \sum_b \alpha(b) F_b^{g0}] \\
&\quad \times [P(g \equiv g_1) F_a^{g1} + (1 - P(g \equiv g_1)) \sum_b \alpha(b) F_b^{g1}] \text{ if g is an internal node.}
\end{aligned}
$$

Computation of $\Pi_a^g = P(g = a \mid \Phi)$

$$
\begin{aligned}
\Pi_a^g &= P(g = a \mid \Phi) \\
\Pi_a^g &= \frac{P(g = a) P(\Phi \mid g = a)}{P(\Phi)} \quad \text{Bayes theorem} \\
\Pi_a^g &= \frac{P(g = a) P(\Phi \mid g = a)}{\sum_b P(R = b) P(\Phi \mid R = b)} \qquad (2)
\end{aligned}
$$

$$
P(\Phi \mid g = a) = P(\Phi_g \mid g = a) P(\Phi - \Phi_g \mid g = a)
$$

$\Phi - \Phi_g$ is the set of leafs that are not in A_g. As the evolution process is reversible, $P(\Phi - \Phi_g \mid g = a)$ is computed as if the root of $\Phi - \Phi_g$ was g.

$$
\begin{aligned}
P(\Phi - \Phi_g \mid g = a) &= \sum_{\theta_g = a_1, \ldots, a_{l(g)} h_g = b_1 \ldots b_{l(g)}} \prod_{i=1}^{l(g)} P(\theta_g^i = a_i \mid \theta_g^{i-1} = a_{i-1}) \\
&\quad \times P(h_g^i = b_i \mid \theta_g^i = a_i) \\
&\quad \times P(\Phi_{h_g^i} \mid h_g^i = b^i)
\end{aligned}
$$

Notations are indicated on Figure 4. $l(g)$ is the number of nodes between g and the root. The summation is taken from the leaf to the root. $P(\theta_g^i = a_i \mid \theta_g^{i-1} = a_{i-1})$ can be deduced from edge length and $P(\Phi_{h_g^i} \mid h_g^i = b^i) = F_{b^i}^{h_g^i}$.

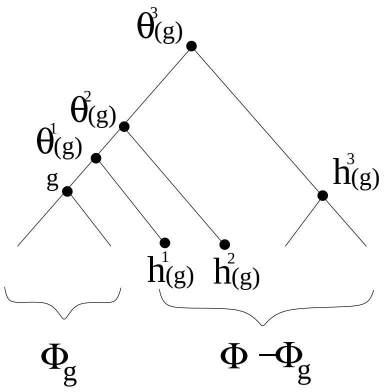

Figure 4: Notations used for the computation of $P(\Phi - \Phi_g \mid g = a)$

1.3.3 Computation of sequence weights

Let consider sequence i with letter a_i. For all the nodes located between the leaf i and the root, with define a coefficient K_i^g:

$$\begin{cases} K_i^g = 1 & \text{if g is a leaf} \\ K_i^g = K_i^{g_{prec}} \frac{I_{a_i}(A_g|\Phi)}{I_{a_i}(A_{g0}|\Phi)+I_{a_i}(A_{g1}|\Phi)} & \text{if g is an internal node} \end{cases}$$

where $K_i^{g_{prec}}$ is the coefficient associated to the node below g.

The final weight associated to sequence i is the coefficient associated to the root.

$$w_i = K_i^R$$

Weights are normalized to sum to 1.

The computation of *real frequencies* involves the use of renewal distribution π. π is the stationary distribution. In this context, it can be chosen as:

1. the observed relative frequency of amino-acids in proteins.
2. distinct laws specific of secondary structures, estimated by the observed amino-acid frequencies. Several weights are then computed and will be used to combine the related posterior probabilities.

The probability of gap in π is taken equal to 5%.

2 Direct coupling of phylogenetic tree with HMM

The idea is to replace in the *forward/backward* algorithm, $b_u(x_t)$, the emission probability of amino-acid x_t in state u, by $b_u(family_t \mid A)$, the probability of observing the given phylogenetic tree A and its leafs, in state u

The computation of $b_u(family_t \mid A)$ is similar to the *real frequencies* computation. This approach is related to the PASSML method [3]. except that the phylogeny is considered to be known.

The computation of $b_u(family_t \mid A)$ is done as introduced by Felsenstein [1].

For each amino-acid a and each node in the tree, we compute the probability to observe the leafs of A_g, given the letter present in g. If g is a leaf:

$$P(\Phi_g \mid g = a) = 1_{\{g=a\}}$$

If g is an internal node:

$$P(\Phi_g \mid g = a) = \left(\sum_{x\in\mathcal{X}} P(\Phi_{g_0} \mid g_0 = x)\left[(1 - P(g \equiv g_o))b_u(x) + 1_{\{a_0=x\}}P(g \equiv g_o)\right]\right)$$
$$\times \left(\sum_{y\in\mathcal{X}} P(\Phi_{g_1} \mid g_1 = y)[(1 - P(g \equiv g_1))b_u(y) + 1_{\{a_0=y\}}P(g \equiv g_1)]\right)$$

with $P(g_0, x) = P(g_0 \equiv g \mid g = a, \Phi_{g_0})$

As the letter of the root is unknown, the sum is taken over all possible amino-acids:

$$b_u(family_t \mid T) = \sum_{a\in\mathcal{X}} b_u(a)P(\Phi \mid g = a)$$

Sequences with gaps are not considered.

3 Simulation studies

Sequence families are simulated from given phylogenetic trees and hidden Markov models.

First, a path in the HMM is generated. An ancestral protein sequence is then simulated conditionally to the path. Sequences of the leafs are then generated given a phylogenetic tree. Edge lengths are expressed in number of mutations for 100 residues. When a mutation occurs, the new amino-acid is generated according to the emission parameters of the corresponding hidden state.

All sequences share the same path.

4 Results

4.1 Comparison of different approaches on real sequences

Results obtained on the independent test set of 505 sequences and OSS-HMM are reported in Table 1.

The model was trained on KAKSI assignments. PSI-BLAST search is done with an e-value threshold of 10^{-3} and the maximum number of iterations is 5. Sequence families are then filtered to remove sequences with less than 30% identity with the query and/or having more than 20% gaps (in the paper sequences are not filtered).

Table 1: Comparison of different approaches to integrate evolutionary information for secondary structure prediction

Method	Q_3 score
Henikoff weighting	75.1 %
Thompson weighting	75.1 %
Equitable sharing weights	74.8%
Direct coupling	65.3%

For the equitable sharing weights, similar results were obtained with one or several novelty laws.

The direct coupling of phylogenetic trees with HMM decreases the prediction scores. This method was then tested on simulated data set in order to:

1. check that the implementation is correct,
2. test the performance in the ideal case where all hypotheses regarding sequence properties are respected.

4.2 Results on simulated data sets

Sequences were simulated with different models:

- a three state HMM (one state per secondary structure),
- the optimal 36 state HMM,
- a 75 state HMM (25 states per secondary structure).

Simulation from artificial phylogenetic trees

We first consider a very basic phylogenetic tree with two sequences, with various distances from the root. In each case 500 sequence families, of length 200, are generated with different models. Predictions are then done using sequence weighting and direct coupling.

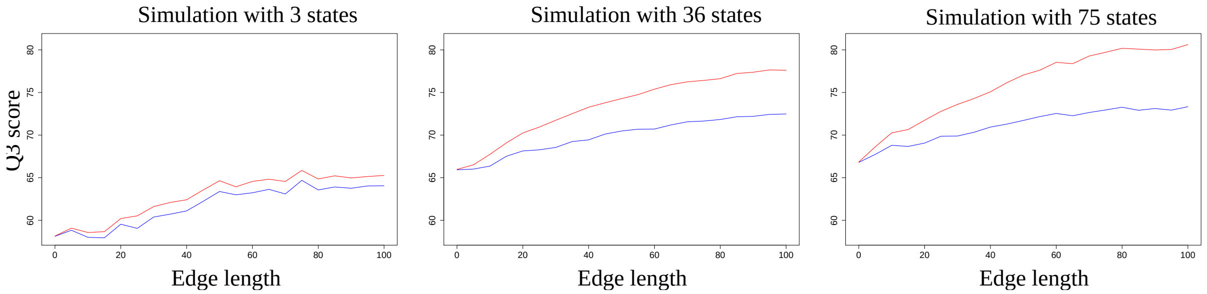

Figure 5: Q_3 score as a function of the edge length. Sequence families are simulated under different HMMs. Blue: scores achieved by sequence weighting, red: scores achieved with the direct coupling.

Direct coupling always gives better results than sequence weighting. The best results are obtained with the largest models. This was expected: a richer model gives a greater contrast between hidden states and thus allows a better prediction relative to a simpler one that can only simulate global behaviours.

Simulation from real phylogenetic data

The central hypothesis of the direct coupling approach is the existence of a common state path of hidden states for all sequences in the family. On real sequences, errors in the alignment could results in shifts that alterate path conservation. This is a first explanation of the poor results obtained with direct coupling approach on real data. Another possibility is that the paths are not conserved. Indeed, if we consider a 36 state HMM and a sequence family, one can imagine that the sequences remain in the same secondary structure but adopt different hidden paths. To test the effect on the non-conservation of hidden paths, sequences were simulated and predicted with different models.

The sequence families of our independent datasets were filtered to keep only the sequences having more than 60% identity with the query sequence and less than 20% gaps. 50 small families are selected, i.e., composed of 2 to 10 sequences. Prediction on real sequences gives the following results:

- using the optimal 36 state HMM (OSS-HMM), $Q_3 = 73.4\%$ with Henikoff weights and $Q_3 = 68.7\%$ with direct coupling.
- using a 3 state HMM, $Q_3 = 62.4\%$ with Henikoff weights and $Q_3 = 62.5\%$ with direct coupling.

Sequence families of length 200 are then simulated using these phylogenetic trees and several HMMs, and the prediction is done with different models and different methods. Results are shown in Table 2. The direct method gives better prediction results when the model used for prediction is similar or less complex (i. e. less hidden states) than the model used for simulation. The use of a more complex model than the one used for simulation systematically results in poor results with the direct coupling approach. Similar results are obtained with larger data sets (data not shown).

When sequences simulated with a 3 state models are predicted with a 36 state model, the hypothesis of path conservation is false: the secondary structure is conserved accross the family, but nothing is known about the conservation of paths in 36 state HMM.

Our simulation studies show that the hypothesis of path conservation is very important for the success of the direct coupling method. This hypothesis may be too strong for real protein sequences.

Table 2: Q_3 scores obtained on a set of 50 simulated protein families

Model used for **prediction**		Model used for **simulation**: 3 state HMM	36 state HMM	75 state HMM
3 state HMM	Henikoff weights	65.8%	64.3%	63.4%
	direct coupling	**71.7%**	**69.0%**	**66.2%**
36 state HMM	Henikoff weights	**59.5%**	73.8%	72.3%
	direct coupling	58.72%	**83.5%**	**75.9%**
75 state HMM	Henikoff weights	**59.1%**	**72.6%**	73.7%
	direct coupling	49.9%	69.6%	**87.0%**

References

[1] J. Felsenstein. Evolutionary trees from dna sequences: a maximum likelihood approach. *J. Mol. Evol.*, 17(6):368–376, 1981.

[2] S. Henikoff and JG. Henikoff. Position-based sequence weights. *J Mol Biol*, 243(4):574–8, 1994.

[3] P. Lio, N. Goldman, J. L. Thorne, and D. T. Jones. Passml: combining evolutionary inference and protein secondary structure prediction. *Bioinformatics*, 14(8):726–733, 1998.

[4] J. D. Thompson, D. G. Higgins, and T. J. Gibson. Clustal w: improving the sensitivity of progressive multiple sequence alignment through sequence weighting, position-specific gap penalties and weight matrix choice. *Nucleic Acids Res.*, 22(22):4673–4680, 1994.
