# Supplementary material for: Analysis of an optimal hidden Markov model for secondary structure prediction
Source: BMC Struct Biol. 2006 Dec 13;6:25. doi: 10.1186/1472-6807-6-25 (PMC1769381; doi:10.1186/1472-6807-6-25)
Supplement: Additional file 5 — Classification of protein sequences using paths in the HMM. Attempt to classify protein sequences using their paths in OSS-HMM. [file 1472-6807-6-25-S5.pdf]

Additional file 5 - Classification of protein sequences using paths in the HMM

An interesting application of our HMM is its use for sequence classification. We present here a preliminary study about the clustering of all-β sequences.

We only use the 571 proteins of the cross-validation data set that belong to SCOP class “all-beta proteins”. The most probable path is computed with the optimal HMM, conditioned by the real secondary structure. In other words, the HMM was fed with both the protein sequence and the secondary structure sequence, to obtain the most probable path given the real secondary structure.

The paths are classified using two types of distances:

1. a distance based on the path composition,
2. a distance based on a definition of the complexity.

For the first distance, each path is converted to a 36-component vector encoding the relative frequency of the 36 hidden states in the path. The distance between two paths is then given by the sum of the absolute deviations between two vectors. Unfortunately, this kind of distance discards a lot of information because paths are reduced to frequencies.

The second distance is based on Kolmogorov complexity. The Kolmogorov complexity of a sequence (Information complexité et hasard, Jean-Paul Delahaye,1999, Hermès Science) is a measure of its information content. It is strongly related to compressibility: the least compressible a sequence, the highest its information content. Kolmogorov complexity is not directly computable, but can be approximated by the length of the compressed file. The distance between two sequences is defined as :

$$d(p_1, p_2) = 2g(p_1 + p_2) - g(p_1) - g(p_2).$$

$p1 + p2$ is the concatenation of the two paths and $g(p)$ is the length of file p after compression.

We used the bzip2 tool because, unlike gzip, it ensures that $g(p1 + p2) = g(p2 + p1)$.

The 571 sequences are then submitted to a hierarchical classification and we confront the resulting paths classification with the fold classification provided by SCOP.

The 571 all-beta sequences belong to 91 different folds. We simplify this classification into 8 main groups of folds: SANDWICH=folds[48725, 49379, 49451, 49492, 69188, 49497, 81278, 49502, 110110, 49561, 49598, 49605, 82003, 88632, 49694, 49722, 49757, 101575, 89231, 63696, 49763, 100919, 49771, 49776, 63706, 63711, 49784, 49817, 82025, 101595, 101600, 101605, 49829, 110131, 49834, 49841, 49853, 49862, 63723, 49869, 49878, 49888, 49893, 49898, 49993, 63736, 50011, 82045, 50016, 50021, 82050], BARREL=folds[50036, 50128, 50155, 50175, 50181, 101737, 101743, 101750, 50192, 50198, 50345, 50352, 50412, 50464, 50474, 69278, 101800, 50485, 50493, 101815, 50609, 50614, 74981, 50629, 50676, 69286, 50684, 50714, 50722, 50728, 50783, 50788, 50799, 50808, 89359, 101851, 100938, 82152, 50813, 50875, 50890, 50903, 63816, 50910, 50915], PROPELLER=folds[50922, 50933, 50938, 50964, 50997], OTHERS=folds[51010, 51044, 51063, 110303, 63839, 101935, 101940, 101998, 69359, 51229, 51268, 82214, 51337, 51343, 81625, 89441, 89446, 88696, 102030, 63886], SINGLE=folds[51068, 89391, 51080, 51086], PRISM=folds[51091, 51109], SUPER-HELIX=folds[51125, 51160, 51181, 69348, 51224], COMPLEX=folds[89427, 89432, 51293, 51305, 69368, 51315, 51321, 82219, 63876, 51326, 51331, 63881],

The resulting classifications are shown on Figure 1, where each protein is shown as a segment colored according to the height main groups. It can be seen that this preliminary paths-based classification correlates poorly with the SCOP classification.

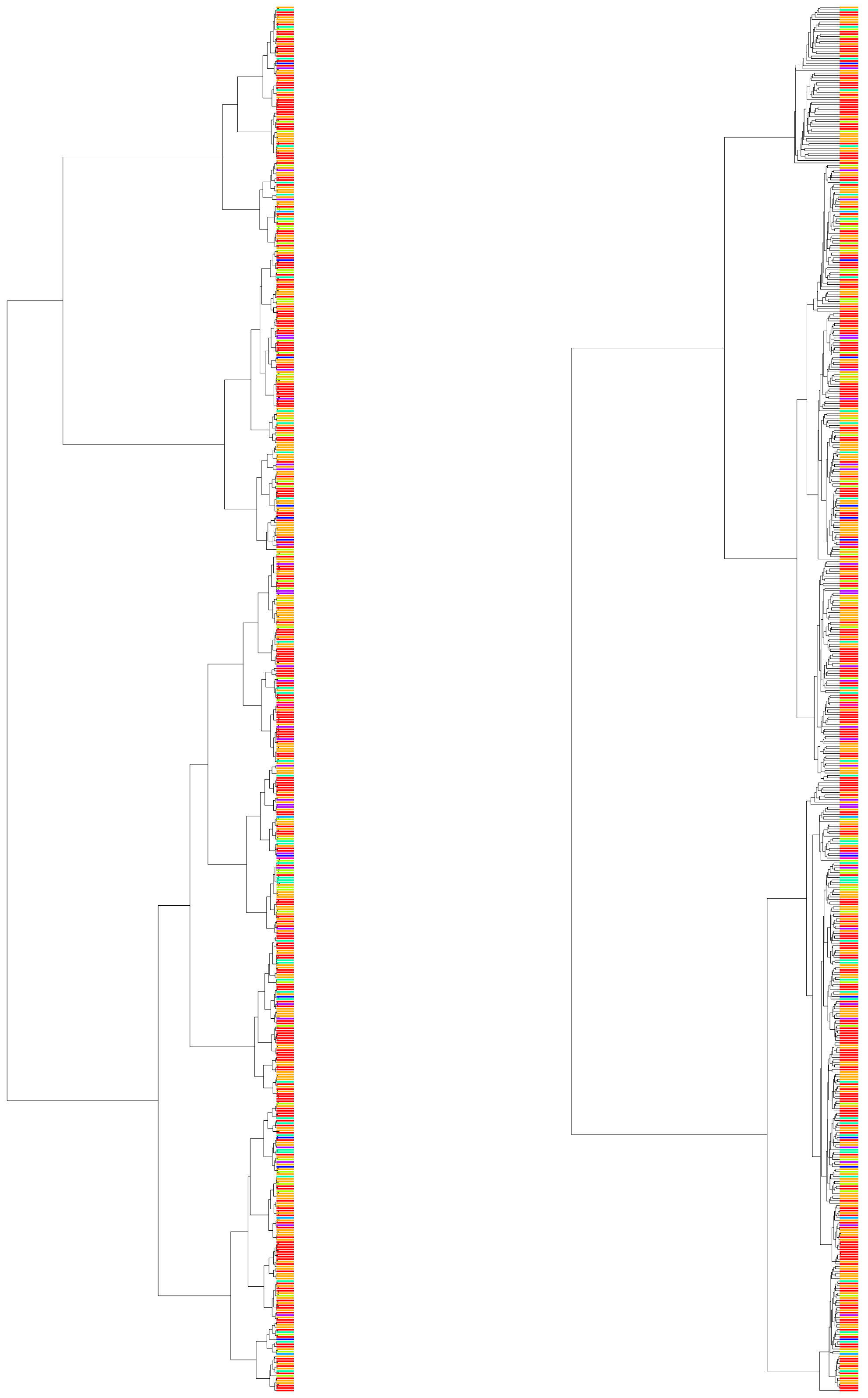

Figure 1: Hierarchical classification of the 571 paths of all-beta proteins. The classification is done using the method "Ward". Each protein is represented by a color segment, whose color correspond to the main group it belongs: orange=BARREL, red=SANDWICH, magenta=PRISM, purple=PROPELLER, marine blue=COMPLEX, blue=SINGLE, blue green=SUPER-HELIX, green=OTHER. Left part: classification using a distance based on state frequencies, Right part: classification using a distance based on the complexity.
