# Supplementary material for: Analysis of an optimal hidden Markov model for secondary structure prediction
Source: BMC Struct Biol. 2006 Dec 13;6:25. doi: 10.1186/1472-6807-6-25 (PMC1769381; doi:10.1186/1472-6807-6-25)
Supplement: Additional file 6 — URL to retrieve the domain lists. Url to retrieve the cross-validation and test data sets. [file 1472-6807-6-25-S6.pdf]

Additional file 6 - URL to retrieve the domain lists

The domain lists used in this study can be retrieved at the following url:
`http://migale.jouy.inra.fr/mig/mig_fr/servlog/oss-hmm`
